# Growth and genome-based insights of Fe(III) reduction of the high-temperature and NaCl-tolerant *Shewanella xiamenensis* from Changqing oilfield of China

**DOI:** 10.3389/fmicb.2022.1028030

**Published:** 2022-12-05

**Authors:** Jiani Yang, Dan Zhao, Tao Liu, Shuang Zhang, Weidong Wang, Lei Yan, Ji-Dong Gu

**Affiliations:** ^1^Heilongjiang Provincial Key Laboratory of Environmental Microbiology and Recycling of Argo-Waste in Cold Region, College of Life Science and Biotechnology, Heilongjiang Bayi Agricultural University, Daqing, China; ^2^Key Laboratory of Low-Carbon Green Agriculture in Northeastern China, Ministry of Agriculture and Rural Affairs, Daqing, China; ^3^Environmental Science and Engineering Research Group, Guangdong Technion – Israel Institute of Technology, Shantou, Guangdong, China; ^4^Guangdong Provincial Key Laboratory of Materials and Technologies for Energy Conversion, Guangdong Technion – Israel Institute of Technology, Shantou, Guangdong, China

**Keywords:** *Shewanella xiamenensis*, Fe(III) reduction, high-temperature, NaCl tolerance, oilfield wastewater

## Abstract

**Introduction:**

A facultative anaerobe bacterium *Shewanella xiamenensis* CQ-Y1 was isolated from the wastewater of Changqing oilfield in Shaanxi Province of China. *Shewanella* is the important dissimilatory metal-reducing bacteria. It exhibited a well potential application in biodegradation and bioremediation.

**Methods:**

Genome sequencing, assembling and functional annotation were conducted to explore the genome information of CQ-Y1. The effect of temperatures and NaCl concentrations on the CQ-Y1 growth and Fe(III) reduction were investigated by UV visible spectrophotometry, SEM and XRD.

**Results:**

Genomic analysis revealed its complete genome was a circular chromosome of 4,710,887 bp with a GC content of 46.50% and 4,110 CDSs genes, 86 tRNAs and 26 rRNAs. It contains genes encoding for Na^+^/H^+^ antiporter, K^+^/Cl^−^ transporter, heat shock protein associated with NaCl and high-temperature resistance. The presence of genes related to flavin, Cytochrome *c*, siderophore, and other related proteins supported Fe(III) reduction. In addition, CQ-Y1 could survive at 10% NaCl (w/v) and 45°C, and temperature showed more pronounced effects than NaCl concentration on the bacterial growth. The maximum Fe(III) reduction ratio of CQ-Y1 reached 70.1% at 30°C without NaCl, and the reduction reaction remained active at 40°C with 3% NaCl (w/v). NaCl concentration was more effective than temperature on microbial Fe(III) reduction. And the reduction products under high temperature and high NaCl conditions were characterized as Fe_3_(PO_4_)_2_, FeCl_2_ and Fe(OH)_2_.

**Discussion:**

Accordingly, a Fe(III) reduction mechanism of CQ-Y1 mediated by Cytochrome *c* and flavin was hypothesised. These findings could provide information for a better understanding of the origin and evolution of genomic and metabolic diversity of *S. xiamenensis*.

## Introduction

The genus of *Shewanella* was discovered by MacDonell and Colwell in 1985 based on 5S rRNA sequences, since then at least 67 species belonging to *Shewanella* have been reported. *Shewanella* spp. usually thrives in the marine, freshwater and spoiled food, but few species could thrive ingeniously in oilfield wastewater with strong corrosion, biodegradability, high temperature and salinity due to their metabolically versatilities (Li et al., 2013; Grouzdev et al., 2018). The species of *Shewanella* isolated from different habitats usually have different physiological and biochemical characteristics.

The vast majority of *Shewanella* from marine environments appeared to tolerate relatively high NaCl concentration (Leblanc et al., 2001). It has been found that some strains of *Shewanella* spp. can survive at the salinity of 7.5% (w/v), e.g., *S. putrefaciens* (Semple and Westlake, 1987; Huang et al., 2010). *Shewanella putrefaciens* mainly performs resistance to osmotic-stress and NaCl through the accumulation of bacterial osmotic active solutes, such as glycine betaine in the cells (Leblanc et al., 2001). Additionally, *Shewanella* spp. usually live in a wide temperature range (4°C–42°C) with an optimal growth temperature around 25°C–28°C. It has been reported that a cold-tolerant strain *S. putrefaciens* from freshwater could grow rapidly and quickly to be the dominant microorganism at 4°C (Yu et al., 2017). It also appeared that *S. algae* could metabolize and grow normally at 42°C, indicating its high-temperature tolerance (Tseng et al., 2018). Recent studies have demonstrated that *Shewanella* spp. could aggregate in the form of biofilms to deal with the harsh environment such as high-temperature and saturated NaCl concentration (Mukherjee et al., 2020). Although the species of *Shewanella* resisted to high temperature or high salinity were obtained easily, there is little information on the member of the *Shewanella* genus from oilfield wastewater exhibiting both high-temperature tolerance and NaCl resistance.

*Shewanella* spp. have attracted great interest due to their diverse anaerobic respiration. As members of facultative anaerobes, they grow by using a variety of substances as terminal electron acceptors (Zinicovscaia et al., 2020). The electrons generated by the bacteria are passed through a specific electron transfer chain and finally onto the electron acceptor for dissimilatory anaerobic respiration and maintain their metabolic growth (Ng et al., 2018). Additionally, *Shewanella* spp. reduce Fe, Mn, Cr and other high-valence metals (Cheng et al., 2021; He et al., 2021). *Shewanella oneidensis*, one species of the most important dissimilatory metal-reducing bacteria (DMRB), is widely used to study the mechanism of electron transfer (Cherkouk et al., 2015).

*Shewanella xiamenensis* is a newly established species isolated from marine sediments in Xiamen. *Shewanella xiamenensis* JCM^T^ 16212, the typical strain of *S. xiamenensis*, could grow in the presence of 0%–4% NaCl (w/v) under 4°C–37°C, but its genomic information has not been reported in detail (Huang et al., 2010). It is a dissimilatory Fe(III)-reducing bacterium that uses the insoluble Fe(III) in the solution as the terminal electron acceptor (Zhao et al., 2020). *Shewanella xiamenensis* exhibited a potential application in biodegradation and bioremediation, although it is considered as a pathogen contributing to abdominal cavity infection (Ma et al., 2018). Since its first isolation, more than 20 genomes of *S. xiamenensis* are available in the NCBI database,^1^ while only one sequence appeared “complete” in the “Assembly level” list, and no description of its growth characteristics has been found (Dao et al., 2022). As a powerful tool, whole-genome sequencing (WGS) can facilitate the deeper insight of Fe(III) reduction performance and mechanism of Fe(III)-reducing bacterium.

In the present study, a new strain of *S. xiamenensis* was successfully isolated from oilfield wastewater and characterized. Genome sequencing, assembling and functional annotation were conducted. The effect of various temperatures and NaCl concentrations on the bacterial growth and Fe(III) reduction were also investigated by UV visible spectrophotometry, SEM (Scanning electron microscope) and XRD (X-ray diffraction). Finally, the hypothetical Fe(III) reduction mechanism of this bacterium were proposed based on genomic annotation and experimental data.

## Materials and methods

### Isolation, 16S rDNA identification, morphology, and siderophore production

The wastewater sample was collected from the Changqing oilfield in Shaanxi Province of China. A 10 ml sample was taken and introduced into a 250 ml conical flask containing 90 ml of sterile Luria–Bertani (LB) liquid medium consisting of (in per liter) 10 g of Na_2_HPO_4_, 5 g of yeast extract powder and 10 g of NaCl (pH 7.0), which was cultured at 30°C for 24 h under shaking condition at 120 rpm (Huang et al., 2010). The 10 ml of bacterial suspension was inoculated into 150 ml conicalflask containing 90 ml LB medium for one subculture. During cultivation, the medium gradually changed from clear to turbid and then to orange color, indicating the presence of bacteria in the culture. After three successive subcultures, the logarithmic-phase cultures were diluted to 10^−8^ by successive 10-fold dilution steps. Taking 100 μl of suspension with dilution from 10^−4^ to 10^−8^ was spread on LB agar plates and incubated at 30°C for 24 h. A single colony was selected and re-cultured and re-spread onto LB agar plates for purification. The isolation process was repeated three times to ensure the purity of the strain. The pure isolate obtained was named CQ-Y1.

Bacterial cells were collected from 1.5 ml of logarithmic-phase cultures by centrifugation at 11,100 × *g* for 1 min. Genomic DNA was isolated from the harvested cells using the DNA midi kit (Biomed, Beijing, China) according to the manufacturer’s instructions. PCR fragments were obtained by amplification of 16S rRNA gene using 27F (5′-AGAGTTTGATCCTGGCTCAG-3′) and 1492R (5′-GGTTACCTTGTTACGACTT-3′) primers. Each 25 μl PCR mixture contained 1.0 μl of template DNA (~50 ng), 1.0 μl of each primer (10 μM), 12.5 μl of 2× PCR master Mix (Biomed, Beijing, China) and 9.5 μl of ddH_2_O. PCR was performed on the 2720 Thermal Cycler Instrument (Applied Biosystems, Foster City, United States) and the PCR followed these steps: 5 min at 94°C, followed by 30 cycles of 94°C for 30 s, 55°C for 45 s and 72°C for 1 min with final extension of 5 min at 72°C. The PCR products were quantified by electrophoresis on 1.0% agarose gel and purified by AxyPrep PCR Clean-up Kit (Axygen Inc., Union City, United States). The gel-purified PCR products were sequenced by BGI (Shenzhen, China). The ChromasPro 2.1.3 software (Technelysium Pty Ltd., Tewantin, Australia) and DNAMAN 6.0 software (Lynnon Biosoft, Quebec, Canada) were used to analyze and assemble 16S rRNA gene contiguous sequences. The nucleotide sequence of 16S rRNA gene was used to find maximum similarity to the bacterial strains in the NCBI database. MEGA11 software (Temple University, Philadelphia, PA, United States) and Clustal W (EMBL, Heidelberg, Germany) were used to perform multiple alignments and the phylogenetic tree construction by the neighbor-joining method (Koichiro et al., 2021). The ANI analysis was performed using JSpeciesWS (Ribocon GmbH, Bremen, Germany) with default settings.

Routine Gram staining reaction was performed and the cells were examined using bright-field microscopy at 100× magnification by using biological microscope Eclipse CI-L (Nikon, Tokyo, Japan). The cell morphology was examined using SEM (Hitachi S-4800, Ibaraki, Japan) and TEM (Hitachi H-7650, Ibaraki, Japan).

The Chrome Azurol S (CAS) two-layer plate method was used to directly detect siderophore according to the previous report (Chen, 1992). In brief, the agar containing the universal blue-colored ferric-CAS complex was used as the lower layer, and the LB medium containing 1.2% agar was used as the upper nutrient layer. The CQ-Y1 was delivered into the plate prepared in advance and incubated at 30°C for 3–4 days. The decolorization of CAS complex and the formation of yellow-orange halo around the upper nutrient layer colonies occurred, once the excreted siderophore penetrates from the upper trophic layer to the lower detection agar. The supernatant obtained from bacterial culture by centrifugation at 11,100 × *g* for 5 min was analyzed by CAS assay to quantify total siderophores (Machuca and Milagres, 2003; Wang et al., 2020). The relative content of the siderophore was calculated by the formula [(Ar − As)/Ar] × 100, where Ar and As are absorption of reference and sample, respectively.

### Genome sequencing, assembling, and functional annotation

The high-quality DNA was extracted by a DNA extraction kit (QIAGEN, Hilden, Germany). The degradation of the samples were detected by agarose gel electrophoresis, DNA purity was detected by Nanodrop, and DNA was accurately quantified by Qubit to detect DNA samples. After the DNA samples passed the quality inspection, the BluePippin automatic nucleic acid cutter was used to recover DNA with a specific fragment size, and then the DNA was damaged and repaired and end repaired. After purification of the magnetic beads, the NBD103 and NBD114 kits (Nanopore, Oxford, England) were used to connect barcode tags to the DNA ends, and the sequencing adapters included in the kits were connected. Finally, Qubit was used to accurately quantify the constructed DNA library. The complete genome sequence of CQ-Y1 was performed on PromethION (Oxford Nanopore Technology, Oxford, United Kingdom). After data quality control, the third generation data were assembled into contigs using the Flye version 2.8,^2^ and the assembled data were rectified by using Racon version 1.4.13^3^ and Pilon version 1.23^4^ to get the final genome sequence.

Coding sequences (CDS) in the genome were predicted through Prodigal version 2.6.3.^5^ The prediction of tRNAs and rRNAs were conducted by tRNAscan-SE version 2.0^6^ and RNAmmer version 1.2.^7^ The gene annotation were carried by Interproscan,^8^ Tigrfams,^9^ Pfam^10^ and GO,^11^ Kegg,^12^ Refseq^13^ and COG^14^ databases. The best results with coverage >30% were retained as the annotation results. The circular map of the genome was obtained using Circos version 0.69.^15^

### Temperature-resistant and NaCl-tolerant experiments

To investigate the high-temperature resistance, *S. xiamenensis* CQ-Y1 was cultured in LB medium whithout NaCl under different temperatures (35°C, 40°C, and 45°C), the incubation at 30°C was used as a control. NaCl tolerance was studied under 30°C using LB liquid medium containing various concentrations of NaCl (2, 4, 6, 8, 10 and 12%, w/v), the control experiment was also performed with the same medium without NaCl at 30°C. All cultures with 10% (v/v) inoculation were incubated on a shaker at 120 rpm for 60 h. To further determine the superimposed effect of high temperature and NaCl concentration on bacterial growth, CQ-Y1 was cultured in LB liquid medium plus different concentrations of NaCl (6, 8 and 10%, w/v) at 40°C and 45°C. The cultures of CQ-Y1 with the same media without NaCl at 40°C and 45°C were used as the control, respectively. The water loss by evaporation in conical bottles was replenished with distilled water by weight method. The bacterial growth for all cultures was monitored every 6 h by measuring OD_600_ values spetrophtometrically. The ability of biofilm formation was assessed by crystal violet assay according to the previous study (De Jesus and Dedeles, 2020). The overnight culture of *S. xiamenensis* CQ-Y1 at different NaCl concentrations and temperatures were dilute 100 times by LB. Added 200 μl of culture to wells of sterile 96-well plates, and incubated at 30°C for 24 h. After the medium was gently sucked out, the bacteria were washed with PBS for 3 times to remove the cells, and then stained with 0.1% crystal violet for 20 min. The crystal violet dye negatively charged molecules and thus stains both bacteria and the surrounding biofilm matrix. The plates were washed exhaustively with distilled water and 200 μl of methanol was added to recover the absorbed stain. Finally, the optical density was measured at a wavelength of 600 nm with an enzyme-labeling instrument (Multiskan GO, Thermo Fisher Scientific, Inc., Rochester, United States). The ability of biofilm formation was assessed by crystal violet assay according to the previous study. The degree of biofilm formation were classified into strong (4 × Ac < A), moderate (2 × Ac < A ≤ 4 × Ac), weak (Ac < A ≤ 2 × Ac), or non-producer of biofilm (A ≤ Ac) based on the absorbance of culture sample (A) and LB medium (Ac) at 600 nm (Pourhajibagher et al., 2016).

### Reduction of Fe(III) under high-temperature and high-NaCl condition

Fe (III) reduction medium consisting of (in per liter) 12.8 g of Na_2_HPO_4_, 3 g of KH_2_PO_4_, 1 g of NH_4_Cl, 2 g of sodium lactate, 2 g of yeast extract powder, and 6.48 g of synthetic Fe(OH)_3_ (pH 7.0) was used to assay the Fe(III) reduction potential of CQ-Y1 (Tor and Lovley, 2001). The effect of various NaCl concentrations (0, 1, 2, 3 and 4%, w/v) or different temperatures (30°C, 35°C, 40°C, 45°C, and 50°C) on Fe(III) reduction were also monitored. The overnight culture of CQ-Y1 were centrifuged at 11,100 × *g* at 4°C for 1 min to harvest bacterial cells, washed twice with phosphate buffer (pH 8.0) and suspended again. A 0.1 ml of bacterial suspension was inoculated into 10 ml serum vial containing 8.9 ml sterile Fe(III) reduction medium. The operation under the same conditions without inoculation were used as control. Each serum vial was flushed with pure N_2_, tightly sealed with stopper and aluminum crimp, and incubated on an Incubator for 60 h. The culture (1.0 ml) was sampled every 6 h and leached with 4 ml HCl (0.5 mol/L) for 24 h. Then, the Fe(II) concentration was measured by phenanthroline spectrophotometry (Han et al., 2018). The Fe(III) reduction ratio was calculated with the equation [C_2_/C_1_] × 100, where C_2_, C_1_ are the Fe(II) concentration in the reaction system and the Fe(III) concentration of the medium, respectively.

### Scanning electron microscope and X-ray diffraction characterization

The morphology of precipitated substances at 0 and 120 h under different culture conditions of Fe(III) reduction expriments were observed by SEM. The samples were vacuum-dried in a desiccator for 7–10 days, and observed by SEM (Hitachi S-4800, Ibaraki, Japan) with a voltage of 5.0 kV. The XRD profiles of precipitates at 0, 72 and 120 h under different culture conditions of Fe(III) reduction expriments were examined by a multipurpose X-ray diffractometer (SmartLab SE, Rigaku Ltd., Japan) using Cu and Kα radiation (*λ* = 0.15406 nm). The scans were performed in the 10° ≤ 2*θ* ≤ 80° diffraction angular range at 25°C in steps of 10° with a recording time of 1 min for each step.

### Statistic analysis

All bacterial culture and biochemical assays were performed with at least three independent repeats for each experiment. Data were presented as means ± standard deviations. The SPSS19.0 software (SPSS Inc., Chicago, IL, United States) was used for all data analysis and OriginPro 2018 software (OriginLab, Northampton, MA, United States) were used to draw all figures. The preference of the cell growth and Fe(III) reduction of CQ-Y1 adapted to high NaCl or high temperature was assessed by the regression using OriginPro 2018 software.

### Data deposition

The complete genome sequence of *S. xiamenensis* CQ-Y1 was deposited in the GenBank database under the accession number CP092630.

## Results

### Basic characteristics and phylogenetic analysis of *Shewanella xiamenensis* CQ-Y1

**Figure 1:**
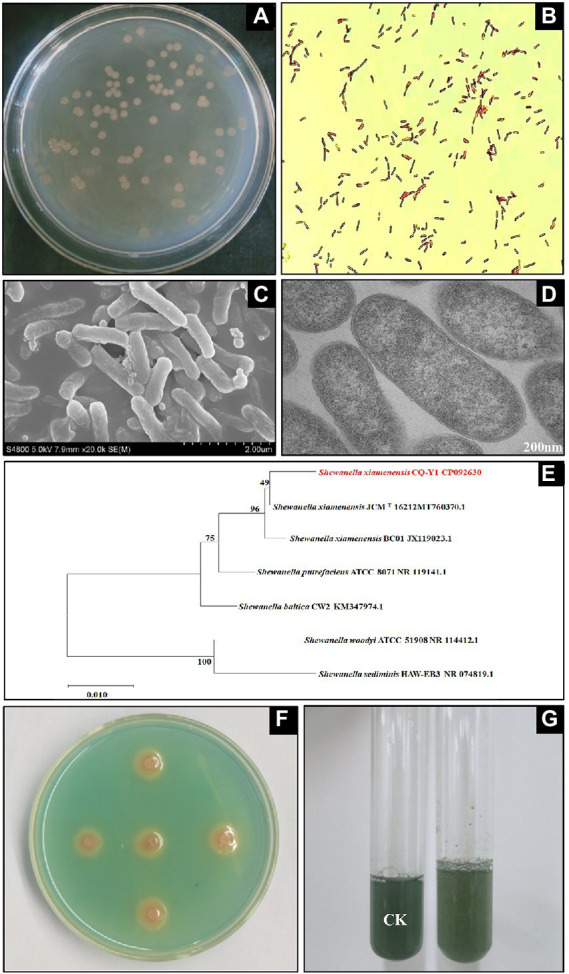
Colony profile **(A)**, gram staining **(B)**, SEM **(C)**, TEM **(D)**, 16S rRNA gene neighbor-joining phylogenetic tree **(E)**, qualitative **(F)** and quantitative **(G)** siderophores determination.

The medium became turbid after incubation of LB medium inoculated with the oilfield wastewater sample at 30°C for 24 h. A large number of colonies appeared on the agar plates after 24 h of incubation at 30°C. Round shaped colonies with light brown color were selected and subcultured in LB liquid medium. A single colony was obtained by repeating the dilution-plate method above-mentioned. The colonies are 2.8 to 3.2 mm in diameter, circular, raised to slightly convex with smooth outline and smooth edges after 36 h incubation (Figure 1A). Gram staining was carried out (Kwon et al., 2019) and the bacterium was Gram-negative (Figure 1B). SEM and TEM showed that the bacterial cells were in rod shape with 0.35–0.46 μm in width and 1.12–1.93 μm in length, and appeared singly or rarely in pairs (Figures 1C,D). The TEM showed that CQ-Y1 have a cell envelope that exhibited the major ultrastructural features of Gram-negative bacteria with intact cell wall and full intracellular contents. The accessory structural of cilia and flagella were not detected in the TEM (Figure 1D).

The partial nucleotide sequence of 16S rRNA gene extracted from the whole genome of CQ-Y1 was used to conduct phylogenetic analysis with the sequences of bacterial strains in the GenBank database. The 16S rRNA gene sequence of CQ-Y1 shares a similarity of 98.96% to *S. xiamenensis* JCM^T^ 16212. The phylogenetic tree constructed by neighbor-joining method of MEGA software shows that CQ-Y1 belongs to *Shewanella* genus (Figure 1E). Additionally, the genomes were compared to confirm the phylogenetic result based on 16S rRNA gene alignment. CQ-Y1 shares the highest ANI value of 96.51% with *S. xiamenensis* NUITM-VS1 (Supplementary Table S1), further indicating that CQ-Y1 closely affiliated to *S. xiamenensis* because strains with ANI ≥ 95% are considered to belong to the same species (Lachance et al., 2020). It could be seen that the clear orange haloes occurred on the double-layer CAS agar plate after 36 h of incubation (Figure 1F), implying that the strain produced siderophores. Using the principle of the color change of CAS blue dye mediated by siderophores, the relative content of siderophores produced by CQ-Y1 was found to be 29.6% (Figure 1G).

### General genome feature

The whole genome sequencing of CQ-Y1 was completed by Nanopore sequencing technology, and its sequencing depth distribution is shown in Supplementary Figure S1. The complete genome of CQ-Y1 consisted of a 4,710,887 bp in a single circular chromosome with 46.50% G + C content, harboring 4,022 predicted coding sequences (CDSs), 78 tRNAs and 21 rRNAs (9 for 16S rRNA, 6 for 23S rRNA, and 6 for 5S rRNA; Supplementary Table S1; Supplementary Figure S2). It is known that the genome size of *S. xiamenensis* strains is between 4.60 and 5.37 Mb with a G + C content ranging from 46.20% to 46.55% (Supplementary Table S1). The COGs analysis showed that the identified 3,959 encoding proteins could be assigned to 26 different categories (Supplementary Table S2). Among them, 157 were assigned to proteins with known functions, and the remaining 398 were regarded as hypothetical proteins. Of these, 10 proteins have no homologs found in other bacterial genomes, possibly suggesting they may be novel proteins.

It could be seen from Supplementary Table S2 that 328 (8.3%) and 274 (6.2%) genes were assigned to Signal transduction mechanisms (T) and Translation, ribosomal structure and biogenesis (J), respectively. It was also found that 263 genes were designated as the amino acid transport and metabolism (E, 6.6%), 238 genes involved in energy production and conversion (C, 6.0%) and 124 genes related to defense mechanisms (V, 3.1%), suggesting that *S. xiamenensis* CQ-Y1 might be able to adapt to extreme environments through metabolism regulation.

### Genome-based functional annotation

*Shewanella xiamenensis* CQ-Y1 was isolated from oilfield wastewater consisting of groundwater and drilling fluid, which had NaCl concentration of 2.65% (w/v). Therefore, CQ-Y1 might also evolved the potential of NaCl tolerance to deal with such extreme environments. Actually, the genome of *S. xiamenensis* CQ-Y1 harbors the genes encoding Na^+^/H^+^ antiporter, K^+^/Cl^−^ transporter and small molecules of intracellular compatibility which associated with NaCl tolerance, such as *nhaB*, *nhaA*, *chaA*, *trkA*, *kdpD*, *proA*, *gltB*, *betT*, and so on (Supplementary Table S3). A large number of thermotolerant microbes could be detected in the high temperature oilfield wastewater. Genes encoding proteins associated with high-temperature tolerance were also found on the genome of CQ-Y1, such as *dnaK*, *grpE*, *aceE*, *lpdA*, *ackA*, *holC*, *priA*, etc. (Supplementary Table S4). Many genes in the CQ-Y1 genome were identified to encode proteins involved in biofilm formation, such as *fliH*, *flhG*, *hfq*, *cps*, *gumC*, etc. (Supplementary Table S5). Environmental tolerance of microbes could be improved by biofilm formation. Additionally, We found genes encoding flavin compounds and Cytochrome *c* protein could involve in Fe(III) reduction, such as *ribA*, *ribB*, *ribM*, *ribH*, *omcA*, *mtrC*, *mtrA*, *mtrB* and *cymA*, on the genome of CQ-Y1 (Supplementary Table S6). These results indicated that *S. xiamenensis* CQ-Y1 might possess the similar characteristics.

### Temperature and NaCl concentration on the bacterial growth

**Figure 2:**
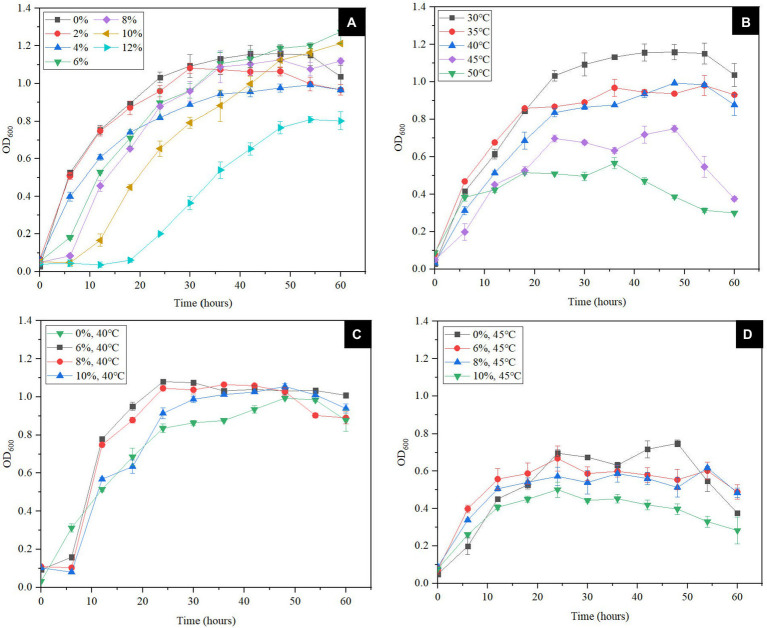
Effects of different NaCl concentrations (0, 2, 4, 6, 8, 10 and 12% w/v) **(A)** and different temperatures (30°C, 35°C, 40°C, 45°C, and 50°C) on bacterial growth **(B)**, effects of different NaCl concentrations (0, 6, 8 and 10% w/v) on bacterial growth at 40°C **(C)** and 45°C **(D)**.

In order to confirm the results of the genomic analysis mentioned above, the tolerance tests were conducted. The degree of NaCl tolerance was determined by the culture of *S. xiamenensis* at different NaCl concentrations (0, 2, 4, 6, 8, 10 and 12%, w/v). As shown in Figure 2A, 2% NaCl (w/v) appeared to have no effect on the growth of CQ-Y1 compared to the control group without any salt, which is consistent with the *S. xiamenensis* strain JCM^T^ 16212 (Huang et al., 2010). CQ-Y1 grows well within a wide NaCl range of 0 to 10% (w/v), although the lag phase was extended by about 6 h when NaCl concentration exceeded 4% (w/v; Figure 2A). The maximum OD_600_ of 1.27 occurred at a 6% NaCl (w/v) concentration, indicating that NaCl within a specific concentration range (0–6%, w/v) could improve the CQ-Y1 growth (Figure 2A). However, the growth of CQ-Y1 was significantly inhibited (*p* < 0.01) when the NaCl concentration reached 12% (w/v). Crystal violet staining assay showed that CQ-Y1 exhibited a high degree of biofilm formation (Supplementary Figures S3A,B) and the biofilm formation ability increased continuously with NaCl concentration from 0 to 12% (w/v) (Supplementary Table S7). CQ-Y1 have exceptional NaCl tolerance when compared with other *shewanella* strains, which might be due to the presence of abundant genes in genome for NaCl resistance (Supplementary Table S8). The growth and metabolism of CQ-Y1 were not inhibited when the NaCl concentration increased to 2% (w/v).

At present, most studies on the temperature tolerance of *Shewanella* spp. only focus on its cold tolerance. Here, we tested the high-temperature tolerance of CQ-Y1 from the oilfield wastewater. The results indicated that the OD_600_ of *S. xiamenensis* reached about 1.1 after 30 h incubation at 30°C, but showed no significant change with increasing temperature from 30°C to 40°C. However, the bacterial growth declined sharply once the temperature reached 45°C, and the maximum OD_600_ of bacterial culture at 45°C reached only half of that at 30°C (Figure 2B). As shown in Supplementary Table S9, the biofilm formation ability was enhanced with the rise of temperature from 30°C to 45°C. Once the temperature was above 45°C, the cell growth was severely inhibited and the biofilm formation significantly weaken (*p* < 0.01; Supplementary Figures S3C,D).

The superimposed effect of high temperature and NaCl concentration on bacterial growth and biofilm formation were further examined. As shown in Figure 2C, the growth of *S. xiamenensis* CQ-Y1 was not significantly affected by the NaCl in the concentration range of 0–10% (w/v) at 40°C. However, the cell growth was severely suppressed by the increase of NaCl concentration at 45°C (Figure 2D). Because of the foregoing, the maximum NaCl concentration and temperature for bacterial growth were 10% and 45°C, respectively. These results showed that *S. xiamenensis* CQ-Y1 exhibted better NaCl and temperature tolerance than other *Shewanella* spp. (Supplementary Table S10).

Temperature and salinity are considered the major controlling factors in the distribution of most bacteria in natural environment (Wang and Gu, 2005). The relationship of bacterial growth with NaCl concentration and temperature were assessed by regression analysis, respectively. The results showed that bacterial growth were both significantly associated with NaCl concentration (*r*^2^ = 0.923, *p* < 0.01; Supplementary Figure S4A) and temperature (*r*^2^ = 0.954, *p* < 0.01; Supplementary Figure S4D), respectively. The correlation coefficient of the latter exceeded that of the former, and its values increased from 0.923 to 0.992 with temperature in the range of 30°C–45°C (Supplementary Figures S4A–C). This finding indicated that temperature displayed a better impact on bacteril growth of CQ-Y1 than NaCl concentration.

### Reduction performance of Fe(III) under high temperature or/and NaCl condition

**Figure 3:**
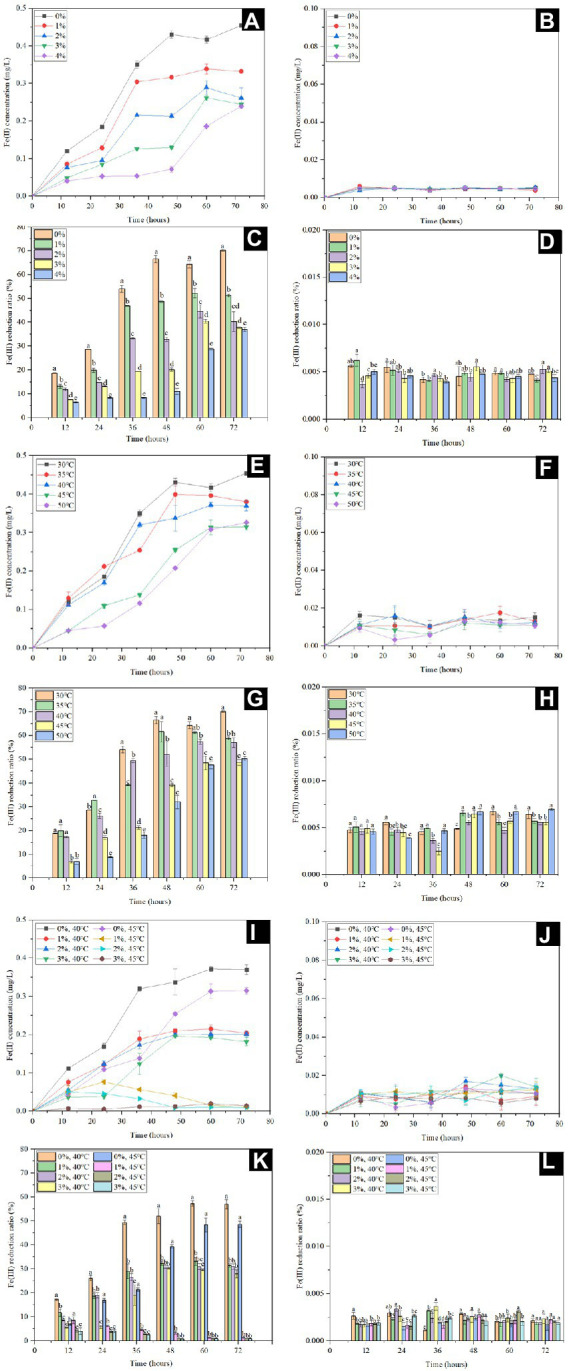
Fe(II) concentration and Fe(III) reduction ratio with **(A,C,E,G,I,K)** and without **(B,D,F,H,J,L)** inoculation at 30°C in the presence of different NaCl concentrations (0, 1, 2, 3 and 4% w/v) **(A,B,C,D)**, different temperatures (30°C, 35°C, 40°C, 45°C, and 50°C) in the absence of NaCl **(E,F,G,H)**, 40°C and 45°C in the presence of different NaCl concentrations (0, 1, 2 and 3% w/v) at **(I,J,K,L)**.

*Shewanella* spp. use FeCl_3_, FeC_6_H_5_O_7_ and Fe(OH)_3_ as the electron acceptor, of which synthetic Fe(OH)_3_ has a high specific surface area (Li et al., 2012). In addition, the suitable electron donors are critical for microbial Fe(III) reduction, and lactate was reported to be the dominant electron donor for *Shewanella* species (Hong and Gu, 2010; Wu et al., 2013). In order to assess the Fe(III) reduction by *S. xiamenensis* CQ-Y1, the Fe(III) reduction experiments were carried out using synthetic Fe(OH)_3_ as substrate and sodium lactate as the electron donor. Figure 3 shows the dynamic variations of Fe(III) reduction at different temperatures (30°C, 35°C, 40°C, 45°C, and 50°C) and NaCl concentrations (0, 1, 2, 3 and 4%, w/v). Fe(II) concentration decreased with an increase of NaCl concenrations at 30°C (Figure 3A), while only trace amounts of Fe(II) were produced without inoculation (Figure 3B). Correspondingly, the reduction ratio of Fe(III) decreased significantly (*p* < 0.05), the minimum value of 36.93% were observed at 4% w/v NaCl (Figure 3C), while the values were <0.01% without inoculation (Figure 3D). Under NaCl-free conditions, the final concentrations of Fe(II) decreased from 0.45 to 0.32 as the temperature increased to 50°C (Figure 3E), but remained low content in the no-inoculation group (Figure 3F). The lowest and highest percentages of Fe(III) reduction (50.3% and 70.1%) were observed at 50°C and 30°C, respectively (Figure 3G), but the reduction ratios were below 0.01% in the control group (Figure 3H). The maximum Fe(III) reduction ratio of *S. xiamenensis* CQ-Y1 was similar to that of *S. oneidensis* (Supplementary Table S10; O'Loughlin et al., 2007).

In order to further understand the maximum potential of Fe(III) reduction under extreme environment, the Fe(III) reduction capacity was determined by the superposition of high NaCl concentration (0, 1, 2 and 3%, w/v) and temperature (40°C and 45°C). The final Fe(II) concentration decreased from 0.37 to 0.18 mg/L with increasing NaCl concentration at 40°C (Figure 3I) and the Fe(III) reduction ratios decreased to 27.99% at 3% NaCl (w/v); (Figure 3K). However, only trace amounts of Fe(II) were produced within 24 h when the NaCl concentration did not excess 2% (w/v) at 45°C, and the Fe(II) was barely detectable after further incubation (Figure 3I). Meanwhile, the Fe(III) reduction ratio of CQ-Y1 were significantly inhibited (*p* < 0.01; Figure 3K). Only trace amounts of Fe(II) were detected in Fe(III) reduction incubation without inoculation (Figure 3J) and the reduction ratios were below 0.01% (Figure 3L).

**Figure 4:**
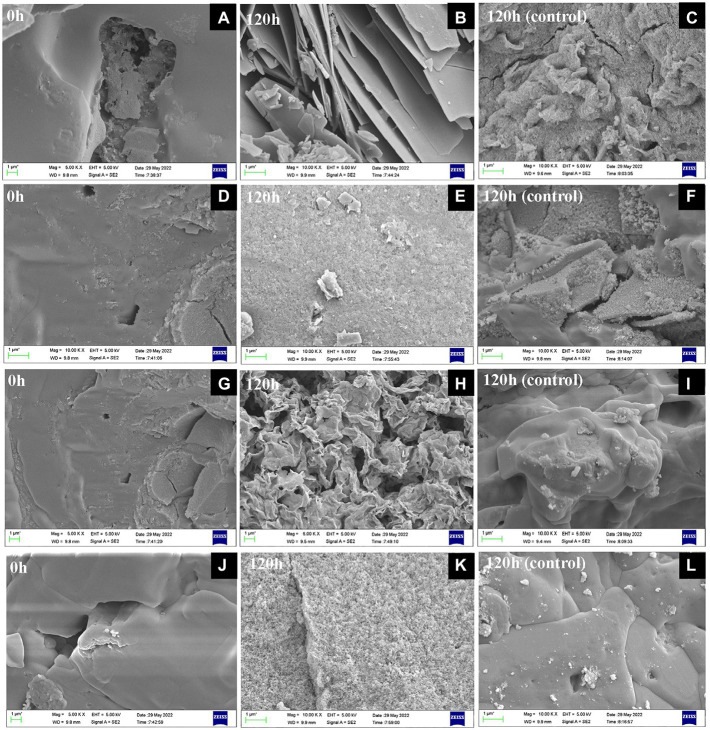
SEM observation of substances after Fe(III) reduction for 0 h **(A,D,G,J)** and 120 h with **(B,E,H,K)** and without **(C,F,I,L)** inoculation at 30°C in the absence of NaCl **(A,B,C)**, 30°C in the presence of 4% w/v NaCl **(D,E,F)**, 40°C in the absence of NaCl **(G,H,I)**, 40°C in the presence of 3% w/v NaCl **(J,K,L)**.

The precipitated substances changed from brown to gray green or white during Fe(III) reduction by *S. xiamenensis* CQ-Y1. The substances after 0 and 120 h were collected and mineralogically characterized using SEM. Under NaCl-free conditions at 30°C, it was observed that the substance presented smooth surfaces with small conglomerates at the starting of experiment (Figure 4A), which is the Fe(OH)_3_ (Carrasco et al., 2021; Zhan et al., 2021). A large number of lenticular shape and plate-like structure, similar to Fe_3_(PO_4_)_2_, were observed at 120 h (Figure 4B). In contrast, the formation of grainy surface were observed Figure 4C probably due to NH_4_FePO_4_ formation in the control group (An et al., 2011). The substance under high NaCl concentration (4%, w/v) condition changed from a smooth surface at the beginning (Figure 4D) to a spongy microstructure at 120 h, which was analog to the structure of FeCl_2_ (Figure 4E; Andrés et al., 2013), while the control was consistent with the substance at 30°C under NaCl-free conditions (Figure 4F). Under NaCl-free condition at 40°C, the smooth form of Fe(OH)_3_ were observed at 0 h (Figure 4G), and the large-scaled film appeared at 120 h, which might be the Fe(OH)_2_ precipitates (Figure 4H; Ma et al., 2013). No significant changes were observed in the control group (Figure 4I). Additionally, we monitored the sbustance at 0 and 120 h during incuabatrion under the superimposed effect of high temperature and NaCl. The smooth substance (Figure 4J) tend to form a spongy microstructure similar to FeCl_2_ (Figure 4K) at 120 h, while the control group did not change significantly (Figure 4L).

**Figure 5:**
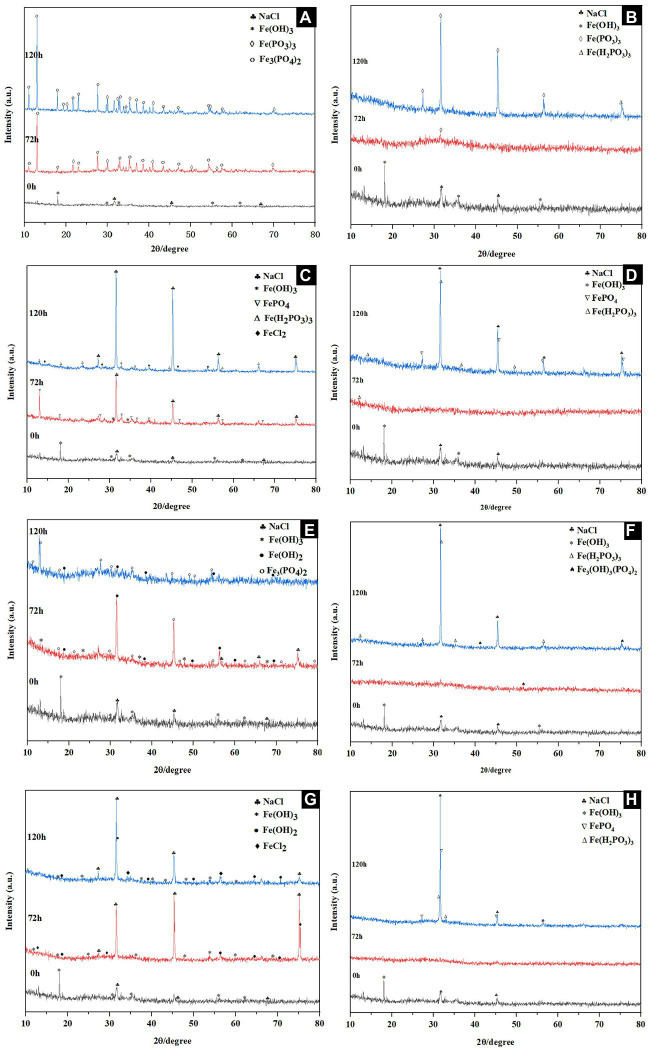
XRD patterns of substances during Fe(III) reduction with **(A,C,E,G)** and without **(B,D,F,H)** inoculation at 30°C in the absence of NaCl **(A,B)**, 30°C in the presence of 4% w/v NaCl **(E,F)**, 40°C in the absence of NaCl **(C,D)**, and 40°C in the presence of 3% w/v NaCl **(G,H)**.

The mineralogical phases and structural properties of products collected at 0, 72 and 120 h under different culture conditions were analyzed with XRD. Fe(OH)_3_ and NaCl peaks occurred at the beginning, the additional peaks of Fe(PO_3_)_3_ and Fe_3_(PO_4_)_2_ appeared after 72 h, and Fe_3_(PO_4_)_2_ with 2θ peaks 11.0, 13.0, 17.9, 19.2, 21.6, 22.9, 26.5, 27.6, 29.6 were mainly observed at 120 h under 30°C NaCl-free reduction condition (Figure 5A), Fe(III) analogs were mainly observed in the absence of inoculation (Figure 5B). Fe(III) reduction products under 30°C and 4% NaCl (w/v) were determined. It could be observed that the additional peaks of FePO_4_ and Fe(H_2_PO_3_)_3_ appeared after 72 h incubation, three wide 2θ peaks 31.5, 45.2, and 75.1 and several weak 2θ peaks 15.0, 35.3, 46.4, 50.6, and 73.2 were captured at 120 h, which were indexed to the phase of NaCl and FeCl_2_ (Figure 5C). The results showed that the reduced products might contain excessive NaCl in the medium and a small amount of FeCl_2_, while no Fe(II) compounds were observed in the uninoculated group (Figure 5D). The XRD pattern of the substance produced by the reduction of Fe(III) at 40°C was similar to that at 30°C, but additional peaks for Fe(OH)_2_ were found (Figure 5E). Only phosphate analogs could be found when inoculated without bacteria (Figure 5F). The dynamics of the product formation under 40°C and 3% NaCl (w/v) was shown in Figure 5G, the characteristic peaks of 31.5, 45.2 and 75.1 were consistent with PDF No. 05-0628 of NaCl standard diffraction card after 120 h incubation. Additionally, the weak peaks of FePO_4_, Fe(OH)_2_ and FeCl_2_ were captured (Figure 5G), which also did not change in the control (Figure 5H). After 120 h incubation, characteristic peaks of NaCl such as 31.5, 45.2 and 75.1 appeared.

To evaluate the relevance of Fe(III) reduction to NaCl concentration and temperature, a relationship of them was established by regression analysis. Results showed the Fe(III) reduction by CQ-Y1 were significantly negatively correlated to NaCl concentration (*r*^2^ = 0.996, *p* < 0.01; Supplementary Figure S5A) and temperature (*r*^2^ = 0.945, *p* < 0.01; Supplementary Figure S5D). It also could be seen that correlation coefficients of regression curves for Fe(III) reduction at 30°C, 40°C, and 45°C (Supplementary Figures S5A–C) kept almost constant with increasing temperature. These results indicated NaCl concentration conferd more effct on microbial Fe(III) reduction than temperature.

## Discussion

### Basic features of *Shewanella xiamenensis* CQ-Y1

The characteristics of CQ-Y1 are very similar to the type strain *S. xiamenensis* JCM^T^ 16212 isolated from coastal sea sediment of Xiamen in China (Huang et al., 2010). Phylogenetic analysis result showed that CQ-Y1 belongs to *Shewanella* spp. It is worth noting that the ANI between *S. xiamenensis* CQ-Y1 and *S. putrefaciens* ATCC 8071 was found to be 96.45%. A similar finding was reported by Kim et al., in which the ANI values of different species within the same genus were 62%–100% (Kim et al., 2014). The reason might be the occurrence of horizontal gene transfer between species witin the genus of *Shewanella*, which requires further investigation (Cruz and Davies, 2000). As a strategy for importing and utilizing Fe(III), siderophore is widely formed by *Shewanella* species, but its relative content has not been reported (Trindade et al., 2019). The relative content of siderophore produced by CQ-Y1 was 29.6%, which is significantly lower than that of high siderophore-yielding strain *Penicillium chrysogenum* (73%; Chowdappa et al., 2020).

### Genomic analysis of *Shewanella xiamenensis* CQ-Y1

#### Resistance to NaCl

Several biological functions of halophiles and their biomolecules could be affected by the changes of environmental ionic composition, including cellular morphology, growth rate, culture density, enzymatic activity, and protein conformation (Fox-Powell and Cockell, 2018). *Shewanella* spp. widely distributed in the sea and brackish water containing high concentrations of NaCl, once the intracellular NaCl content exceeded a certain threshold level, the adverse effects of the excessive accumulation of Na^+^ would cause hyperosmotic stress, ionic toxicity, and nutritional imbalance to microbial cells (Peng et al., 2021). Therefore, most strains of *Shewanella* genus have evolved the ability to tolerate NaCl for adapting the saline habitat, such as changes in the expression of salt-responsive genes, modifications of lipid composition and the accumulation of osmoprotectants (Leblanc et al., 2001; Kuribayashi et al., 2017). The comparative genome analysis indicated that the genes related to NaCl tolerance existed in CQ-Y1 were also found in genomes of other *Shewanella* spp. (Supplementary Table S8; Zeng et al., 2010).

As one of the mechanisms on regulation of Na^+^ and H^+^ concentrations across biological membranes, Na^+^/H^+^ antiporters mainly confer to maintain stable Na^+^ concentration by coupling the import of H^+^ to outport intracellular excess Na^+^. Many genes encoding Na^+^/H^+^ antiporter members such as the family of Cpa2, Mfs, NhaA, NhaB and NhaC were found in the genome of CQ-Y1. Among them, NhaA is one of the most well studied Na^+^/H^+^ antiporters in microorganisms, deletion of its encoding gene often leads to bacterial growth defects under NaCl stress (Patiño-Ruiz et al., 2019). NhaB and ChaA are also the common Na^+^/H^+^ antiporters in bacteria. Together with the Na^+^/H^+^ exchanger NhaB and ChaA, NhaA allow bacteria to respond to environmental stress (Patiño-Ruiz et al., 2019). In addition, the accumulation of high intracellular concentration of K^+^/Cl^−^ to maintain the osmotic equilibrium of halophilic microorganisms is proposed. Three genes (*trkA*, *trkH*, *kdpD*) involved in K^+^ uptake were found to exist in *S. xiamenensis* CQ-Y1 (Supplementary Table S1), which encod the proteins to balance the external high osmotic pressure through the accumulation of intracellular K^+^ with the same molar concentration (Kraegeloh et al., 2005).

It has been reported that some halophilic microorganisms could absorb, synthesize and accumulate sugars, amino acids, betaine and other intracellular compatible small molecules under high NaCl pressure (Qin et al., 2020). The accumulation of these small molecular weight substances compensates for the imbalance of internal and external osmotic pressure caused by high osmotic pressure outside, and alleviates the stress on cells exposed to high NaCl concentration to a certain extent. Five genes (*proA*, *proB*, *proC*, *gltB*, *gltD*) associated with the production and metabolism of proline were abundant in the *S. xiamenensis* CQ-Y1 genome, which might be beneficial to bacterial resistance to NaCl stress. Additionally, the glycine betaine was also proven to play an essential role in the osmotic adaptation of halophiles (Qin et al., 2020). It has been described that *S. putrefaciens* accumulated glycine betaine from choline to resist hypertonic conditions (Leblanc et al., 2001). The *betT* and *betA* genes encoding the glycine betaine transporter appeared in the genome of CQ-Y1, but how/if these genes and their encoded products play a role in resistance to hyperosmotic stress is unknown. These results suggested that *S. xiamenensis* CQ-Y1 might respond to NaCl stress.

#### Resistance to high-temperature

Bacterial proteins undergo an irreversible degeneration under high-temperature stress, resulting in cell structure damage. However, bacteria are equipped with cell membranes, excess antioxidant proteins, and active energy metabolism against the stress caused by high temperatures (Murata et al., 2011). Membranes can adapt their physicochemical properties (e.g., lipid class, fatty acid chaing length and saturation) to environmental stresses. Generally, bacteria reinforce their cellular membranes by increasing the total lipid content and saturated fatty with a high melting point to resist continuous heat stress (Urbieta et al., 2015). Prokaryotes living at high temperatures exhibit pronounced signatures in the amino acid composition of their proteins (Zeldovich et al., 2007). The enhanced thermostability is reflected in specific trends in amino acid composition, particularly the hydrophobic residues in (hyper)thermophilic organisms are increased. The increase of the content of hydrophobic (Val, Trp, Leu, Arg, Glu) amino acids can enhanced thermostability (Zeldovich et al., 2007). Additionally, GrowthPred found amino acids remarkably positively correlated with optimal growth temperature. The optimal growth temperatures of bacteria could be influenced by GC-rich codons, such as CGC and CGG (for Arg), GAG (for Glu), and CUC and CUG (for Leul Sriaporn et al., 2021). Additionally, exposure to high temperatures could induce the expression of heat shock proteins to protect the cells in most cases, including GrpE, DnaK, DnaJ and other chaperones (Kim et al., 2020). The genes encoding heat shock proteins GrpE, DnaK, DnaJ, and GroEL to participate in the heat shock and hyperosmotic responses were detected in the chromosome of *S. xiamenensis* CQ-Y1. These genes also exist in psychrophilic *S. frigidimarina*, which were characterized by their cold adaptation (Supplementary Table S8; Yoshimune et al., 2005).

DNA reverse gyrase and a high concentration of K^+^ in microbial cells are important factors for maintaining the thermal stability of DNA molecules (Duprey and Groisman, 2021). DNA molecules are easily subjected to double-strand breaks at high temperatures, so cells need some genes for DNA double-strand break repair, including *dnaQ*, *holC*, *priA* and *ruvA* (Lenhart et al., 2012). The binding of K^+^ to DNA molecules can prevent the loss of purines at high temperatures. Generally, the higher the temperature, the more ATP is required for the cells (Murata et al., 2011). Most of the genes related to high-temperature tolerance mentioned above also existed in other thermo-tolerant *Shewanella* spp., suggesting that *S. xiamenensis* CQ-Y1 could survive in high-temperature stress environments (Supplementary Table S8; Vogel et al., 1997; Khashe and Janda, 1998).

#### Biofilm formation

Microorganisms communicate with each other with more sophisticated regulatory mechanisms. As a classic example, biofilms are surface-adhered communities of microbial cells embedded in a self-produced extracellular polymeric matrix. The microorganisms in biofilm have low metabolic activity, long survival time and strong adaptability. *Shewanella* species can form biofilms as one way for adapting to extreme environments (Prasad et al., 2019). Flagella-mediated motility plays an important role in both surface adhesion and biofilm formation (Moe et al., 2010). Genes of *fliH*, *fliI*, *fliJ*, *flhG*, *flhF*, *flhC*, *lafK*, and *filK* harbored in the genome of CQ-Y1 were annotated as encoding flagellum-related proteins, key components of bacterial movement. The *fliC* mutant *Escherichia coli* strain NU149 could not move on the soft agar (Schwan, 2008). The *lafK* gene encodes a protein that regulates the expression of lateral flagella genes, *flhF* and *flhG* genes are associated with the regulation of flagella number, *fliH*, *fliI*, and *fliJ* encode the ATPase complex for selective delivery of transported substrates to the flagellum base to drive motility (Osterman et al., 2015). Although we found flagella-related coding genes in the genome of CQ-Y1, the flagella ultrastructure was not observed in the TEM image, which might be due to the loss of the accessory structural during specimen preparation.

Several genes related to quorum-sensing (QS) system exist in the genome of CQ-Y1, including *luxS*, *hfq*, and *abaI* (Supplementary Table S5). Generally, specific signal molecules would be secreted to activate the bacterial QS system once the bacterial density reached a specific threshold for bacterial biofilm formation (Guo et al., 2018). AI-2 is a signal for intra-species and inter-specific communication, and *luxS* is associated with the synthesis of AI-2 (Guo et al., 2018). The gene *hfq* significantly regulates the transcription of the acylated homoserine lactone (AHL) synthase gene *pcoI* (Wu et al., 2010). And *abaI* gene could regulate the secretion of AHLs (Tang et al., 2020). The QS system can ensure the steady growth of biofilms because it can facilitate the transport of nutrients and discharge waste generation.

Genes related to extracellular polymeric substances (EPS) synthesis, such as *galU*, *cps*, and *gumC*, were also identified in the genome of CQ-Y1. As a housekeeping gene in EPS synthesis, *galU* encodes UGPase, a critical control point in EPS production (Boels et al., 2001). In addition, the *cps* gene cluster encodes nucleotide sugar synthase, acetyltransferase, nucleotide sugar processing enzyme and so on, and the *gumC* forms the unit structure of polysaccharide as a monosaccharide transferase (Kolkman et al., 1997; Vorhölter et al., 2008). As already known, biofilm protects bacterial communities mainly through encasing cells within EPS, suggesting that *S. xiamenensis* CQ-Y1 might have strong biofilm-forming ability.

#### Fe(III) reduction

During the evolution, bacteria have developed the ability to protect themselves from heavy metal toxicity through various mechanisms such as adsorption, oxidation, and reduction (Zinicovscaia et al., 2020). *Shewanella* spp. belonging to dissimilatory metal-reducing bacteria reduce Fe(III), Mn (IV), Se (V) and other multivalent elements (He et al., 2021). Over the past years, although significant progress has been made in molecular understanding of extracellular electron transfer mechanisms during Fe(III) reduction by *Shewanella* species, such as *S. oneidensis* and *S. putrefaciens*, little attention has been devoted to *S. xiamenensis* (Jiang et al., 2020).

Flavin compounds including riboflavin (RF), flavin mononucleotide (FMN) and flavin adenine dinucleotide (FAD) play important roles in the microbial indirect electron transport, which is one of the mechanisms of extracellular electron transfer for microorganisms (Tian et al., 2019). RF is, the direct precursor of the coenzymes FMN and FAD, can be hydrolyzed to FMN which could be extracellularly converted to FAD under the action of phosphatase (Grill et al., 2007). It has been reported that FAD, FMN and RF could act as electron mediators to regulate electron transport between the cell surface and terminal electron receptor (Tian et al., 2019). The genes *ribA*, *ribB*, *ribM* and *ribH* closely related to the synthesis of riboflavin were identified in *S. xiamenensis* CQ-Y1 (Grill et al., 2007), suggesting that CQ-Y1 have the ability to synthesize riboflavin and possibly secrete flavin compounds for indirect electron transfer. This finding agrees with the results from the previous study on *S. oneidensis* MR-1 (Cherkouk et al., 2015).

C-type cytochrome CymA, an essential substance for direct extracellular electron transfer, exists in most *Shewanella* species (Ghasemi et al., 2020; Jiang et al., 2020). Additionally, MtrA, FccA, MtrB, and OmcA proteins play an indispensable role in the electron transport system. The electrons transfer from CymA to MtrA in the periplasm, then to MtrB on the outer membrane, and finally transfer to terminal reductases OmcA and MtrC (Ghasemi et al., 2020). Several protein components involved in the Mtr pathway, including CymA, MtrA, MtrB, MtrC, and OmcA encoded by the sequence *omcA*–*mtrC*–*mtrA*–*mtrB*–*cymA* in the genome of *Shewanella* species (Ng et al., 2018). The genes encoding these proteins were also found on the genome of *S. xiamenensis* CQ-Y1, indicating the possible presence of Mtr pathway. The binding of C-type cytochrome with flavin compounds can enhance the extracellular electron transfer in *Shewanella* (Okamoto et al., 2014), but whether this mechanism exists in *S. xiamenensis* needs further study. These above metioned genes that are widely present in the iron-reducing *Shewanella* spp. might confer to Fe(III) reduction of CQ-Y1.

### High temperature and NaCl resistance of *Shewanella xiamenensis* CQ-Y1

Some *Shewanella* species isolated from the ocean can thrive under 0%–10% NaCl (w/v) conditions (Kuribayashi et al., 2017; Song et al., 2022; Zhang et al., 2022). Simliarly, CQ-Y1 grows well within a wide NaCl range of 0 to 10% (w/v). However, the lag phase of CQ-Y1 was extended by about 6 h when NaCl concentration exceeded 4% (w/v). The extension of the lag phase might be due to the adverse effects of high osmotic stress, ionic toxicity and nutritional imbalance caused by excessive Na^+^ accumulation on microbial cells. Growth of CQ-Y1 could be improved at salt levels of no more than 6%. This might be attributed to the presence of the halophilic enzyme, which could prevent the denaturation and inactivation of bacterial proteins only at high NaCl concentrations (Mariana et al., 2012). Appropriate NaCl concentration could prompt the expression of some genes encoding Na^+^/H^+^ transporters, proline, betaine and other intracellular compatible small molecules to maintain osmotic balance (Qin et al., 2020). Additionally, NaCl within a certain concentration range could stimulate the EPS production and biofilm formation of *S. xiamenensis* CQ-Y1 (Boels et al., 2001). However, once NaCl concentration has reached a critical value or salinity exceeds the tolerance range of CQ-Y1, high concentrations of Na^+^ produce toxicity, leading to impaired metabolism and decreased cellular metabolic activity (Peng et al., 2021).

Although the cold-adapted *Shewanella* spp. are widely found, a few strains can grow at high temperatures (Tseng et al., 2018). The strong tolerance to high temperature stress makes CQ-Y1 stand out in the *shewanella* spp. due to the presence of a large number of high temperature tolerance genes in genome. Tolerance tests result showed that CQ-Y1 could survive at 30°C–45°C. The total lipid content and saturated fat have higher melting points, and the expression of related genes enhances the heat stress resistance of bacteria (Urbieta et al., 2015). Additionally, biofilm plays an important role in the whole process of microbial resistance to high-temperature stress (Prasad et al., 2019). The CQ-Y1 growth was severely inhibited when the temperature was above 45°C. High-temperature stress can cause the irreversible denaturation of bacterial proteins, which would lead to the disintegration of cell structure, resulting in bacterial growth inhibition (Murata et al., 2011). The growth of CQ-Y1 was not significantly affected by the NaCl in the concentration range of 0–10% (w/v) at 40°C. Heterologous stress pretreatment could induce higher resistance toward osmotic challenges. *Shewanella putrefaciens* grown at low temperatures can provide cross-protection against lethal NaCl treatment, and whether a similar mechanism can arise when CQ-Y1 produces mild heat shock remains to be confirmed (Leblanc et al., 2010).

### Reduction performance of Fe(III) of *Shewanella xiamenensis* CQ-Y1

Fe(III) reduction experiments results indicated that CQ-Y1 could still perform Fe(III) reduction under certain NaCl concentrations and/or temperatures. Bacteria tend to aggregate during freezing damage, cold shock and thermal stresses, and the rate of bacterial death could be moderated by higher bacterial densities to some extent (Rong et al., 2022). However, if high bacteria densities sustain for a long time, the organisms would die quickly caused by hypoxia. Similarly, the bacteria died rapidly when the organism was stressed enough to break the osmotic barrier (Konings et al., 2002). CQ-Y1 increased the survival rate by aggregating together, and continued to exert its Fe(III) reducing capacity under a certain stress. The low concentration of Fe(III) could promote the secretion of EPS in the biofilm, which is more conducive to microbial aggregation, external pressure resistance and Fe(III) reduction reaction (Hu et al., 2016). In addition, the chemical speciation governs Fe(III) bioavailability, changes in Fe(III) morphology during reduction might prevent the continuation of Fe(III) reduction (Zhang et al., 1995; Haas and Dichristina, 2002). However, trace amounts of Fe(II) could also be detected in Fe(III) reduction culture without inoculated. It has been reported that these Fe(II) might derived from the substance produced by chemical reactions and ion exchange in the solution, for instance, formation of NH_4_FePO_4_ (An et al., 2011).

Combining the SEM and XRD analysis results, we found that the increase in NaCl concentration was accompanied by the accumulation of chloride ions in solution and the Fe(II) generated by reduction tended to combine with chloride ions rather than phosphate, resulting in a predominance of FeCl_2_. With temperature rises, there is a tendency for Fe(OH)_2_ formation. Observation of the combined effect of high temperature and NaCl concentration on the reduction products revealed that their precipitation tended to form a spongy microstructure similar to FeCl_2_, implying that Fe(II) tended to bind with the excess chloride ions in solution. In the control group, we found the amorphous organization predominated at 72 h, and Fe(III) tended to combine with phosphorous acid radicals to form FePO_4_, Fe(H_2_PO_3_)_3_, Fe_3_(OH)_3_(PO_4_)_2_ and other substances with the time increased to 120 h. Trace amounts of Fe(II) could be detected without inoculation during the determining of Fe(III) reduction ratio of CQ-Y1. However, no Fe(II) compounds were detected in the XRD and SEM results under the identical condition. This suggested that the trace amounts of Fe(II) did not influence the macroscopic product changes and had no contribute to the Fe(III) reduction reaction of CQ-Y1. Furthermore, the Fe(III) reduction ability of *S. xiamenensis* CQ-Y1 was weak under the superimposed effect of high temperature and NaCl concentration. Fe(III) could be reduced to Fe(II), and the Fe(II) combined with PO_4_^3−^ to form a complex similar to Vivianite [Fe_3_(PO_4_)^2^·8H_2_O], indicating the gray-green precipitates might be Fe_3_(PO_4_)_2_ (John et al., 2001). It is generally believed that lower phosphate concentrations are conducive to green rust formation, and green rust is more easily converted into Fe_3_(PO_4_)_2_·8H_2_O at high phosphate concentrations (Li et al., 2012). *Shewanella* spp. can transform hematite to iron oxide hydroxide and iron phosphate (Zhao et al., 2020), and was able to reduce akaganeite to form magnetite (Roh et al., 2006).

### Speculation on Fe(III) reduction mechanism

**Figure 6:**
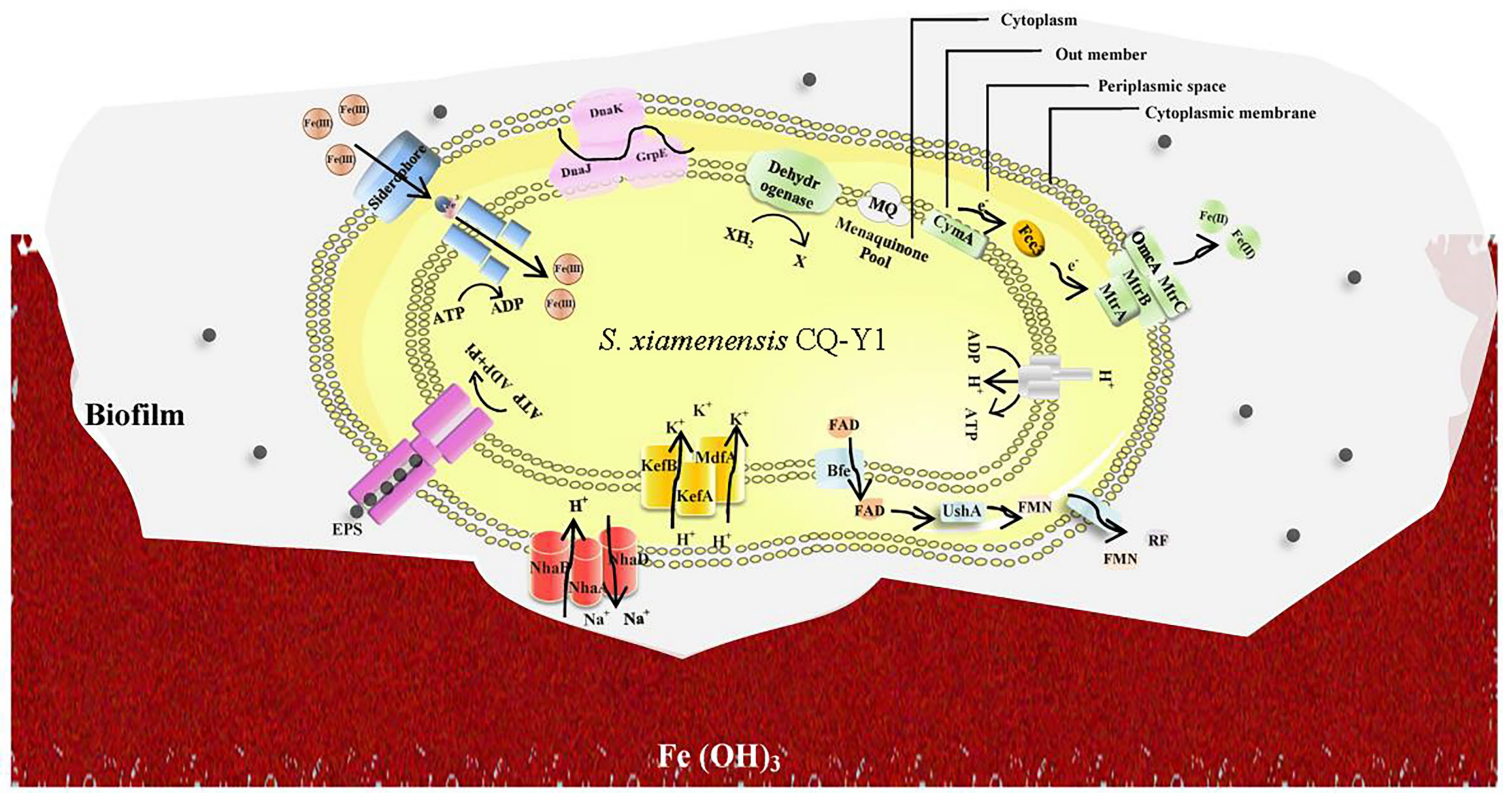
A hypothetical Fe(III) reduction mechanism of *Shewanella xiamenensis* CQ-Y1.

Based on the genomic analysis and Fe(III) reduction performance, a hypothetical Fe(III) reduction mechanism of CQ-Y1 was proposed. As shown in Figure 6, CQ-Y1 might have two main electron transport pathways associated with Fe(III) reduction under favorable conditions. (1) The electrons transfer from the cytochromes around the cytoplasm to the terminal reductases on the cell surface (Ghasemi et al., 2020), and transfer into the quinone pool *via* the NADH carrier, the CymA protein on the inner membrane transfers electrons to the periplasm, Fcc_3_ in the periplasmic space transfers electrons to a transmembrane protein complex of MtrA, MtrB and MtrC on the outer membrane, which in turn transfers electrons to the cell surface, and electrons were transferred directly to iron oxide through the contact of OmcA and MtrC on the cell surface (Sheng et al., 2016). (2) Bacteria transport intracellular FAD to periplasmic space, and then hydrolyze to FMN, which is released into the extracellular environment through pore-forming proteins in the outer membrane and acts as electron shuttle carrier to transport electrons (Grill et al., 2007). In addition, siderophores synthesized intracellularly by bacteria could be secreted to capture Fe(III) in the extracellular environment, enabling them to achieve specific chelation of Fe(III) even under low Fe(III) conditions (Nie et al., 2021).

However, under high temperature and high NaCl conditions, CQ-Y1 might highly express Na^+^/H^+^ antitransporters and K^+^/Cl^−^ transporters to achieve strict regulation of Na^+^ concentration, induce the expression of heat shock proteins to protect the cells, including GrpE, DnaK, DnaJ and other chaperones (Figure 6). At the same time, under such conditions, CQ-Y1 is easy to develop biofilm on iron oxides, which enable more accessible to cell metabolism (Zinicovscaia et al., 2020). The biofilms can confer protection by limiting the access of dissolved oxygen to the metal and releasing intracellular electrons to solid metallic structures (Zinicovscaia et al., 2020). In this process, the QS system can help bacteria adhere and secrete a large number of EPS, which specifically binds to metal ions, promoting Fe(III) reduction reaction (Rong et al., 2022). Once the temperature exceeds 45°C and the NaCl concentration exceeds 10% (w/v), all aspects of the biological function of the bacteria would be damaged (Fox-Powell and Cockell, 2018). The Fe(III) reduction capacity inevitably decreases when cells cannot form biofilms to sustain the nutrients and energy transport needed for normal growth.

Microbially induced corrosion is a complex problem causing many hazards. *Shewanella* spp. are regularly found on corroded metal structures. The corrosion effects of *Shewanella*, a typical iron reducing bacterial genus, on steel have been extensively studied, but the evidence for these effects remains controversial (Robert et al., 2016). It has been suggested that *Shewanella* could reduce insoluble Fe(III) to soluble Fe(II) and consume oxygen, thus, limiting the interaction between O_2_ and the metal surface (Jo et al., 2018). On the other hand, Fe(III) respiration may contribute to the reductive dissolution of the Fe(III) (hydr)oxide protective layer, promoting the detstruction of the steel (Li et al., 2017). The research of biofilms in microbially induced corrosion is also crucial, microbial activity in biofilms may cause changes in chemical conditions. Biofilms may induce corrosion by forming different aeration and concentration cells on metal surfaces (Robert et al., 2016). This information can be used to develop preventive methods and monitoring tools for microbially induced corrosion.

## Conclusion

The complete genome of *S. xiamenensis* CQ-Y1 isolated from wastewater of Changqing oilfield in Shaanxi of China is consisted of a chromosome with 4,710,887 bp. The CQ-Y1 has abundant function genes related to NaCl resistance, high-temperature adaptation and Fe(III) reducing capacity. It could survive under 0–10% NaCl (w/v) and at 30°C–45°C. The maximum Fe(III) reduction ratio of it reached 70.1% and reduction reactions was still active under 40°C and 3% NaCl (w/v). The bacterial growth appeared more sensitive to temperature than NaCl concentration, the inverse effects on Fe(III) reduction were observed. SEM and XRD revealed that Fe(OH)_3_ was reduced to the mixture of Fe_3_(PO_4_)_2_, FeCl_2_ and Fe(OH)_2_ by CQ-Y1. A hypothesized Fe(III) reduction mechanism consisting of both Cytochrome *c* and flavin is proposed. These findings would contribute to further study on the extreme environmental tolerance, Fe(III) reduction and corrosion mechanism of *S. xiamenensis*.

## Data availability statement

The datasets presented in this study can be found in online repositories. The names of the repository/repositories and accession number(s) can be found in the article/Supplementary material.

## Author contributions

LY contributed to conceptualization and validation, investigation, and funding acquisition and performed project administration. JY performed writing–original draft preparation. JY, J-DG, and DZ performed writing–reviewing and editing. WW and TL provided methodology. SZ performed data curation and visualization. J-DG was involved in supervision. All authors contributed to the article and approved the submitted version.

## Funding

This work was supported by the Talent Training Program under Special Funds Supporting the Heilongjiang Provincial Key Research and Development Program Guidance Projects (GZ20220051), the Development of Local Universities from the Central Finance (HFBE[2019]465), Heilongjiang Provincial Natural Science Foundation of China (LH2020C079), Longjiang Scholar Program of Heilongjiang Province (Q201815), Strategic Priority Research Program of the Chinese Academy of Sciences (XDA28030203), Heilongjiang Bayi Agricultural University Support Program for San Heng San Zong (ZRCQC202206).

## Conflict of interest

The authors declare that the research was conducted in the absence of any commercial or financial relationships that could be construed as a potential conflict of interest.

## Publisher’s note

All claims expressed in this article are solely those of the authors and do not necessarily represent those of their affiliated organizations, or those of the publisher, the editors and the reviewers. Any product that may be evaluated in this article, or claim that may be made by its manufacturer, is not guaranteed or endorsed by the publisher.

## Supplementary material

The Supplementary material for this article can be found online at: https://www.frontiersin.org/articles/10.3389/fmicb.2022.1028030/full#supplementary-material

## Footnotes

^1^ https://www.ncbi.nlm.nih.gov/

^2^ https://github.com/fenderglass/Flye

^3^ https://github.com/isovic/racon

^4^ https://github.com/broadinstitute/pilon

^5^ https://github.com/hyattpd/Prodigal

^6^ http://lowelab.ucsc.edu/tRNAscan-SE

^7^ http://www.cbs.dtu.dk/services/RNAmmer

^8^ http://www.ebi.ac.uk/interpro/interproscan.html

^9^ http://tigrfams.jcvi.org/cgi-bin/index.cgi

^10^ https://pfam.xfam.org/

^11^ http://geneontology.org/

^12^ https://www.kegg.jp/kegg/

^13^ https://www.ncbi.nlm.nih.gov/refseq/

^14^ https://www.ncbi.nlm.nih.gov/COG/

^15^ http://circos.ca
