# Supplementary material for: Growth and genome-based insights of Fe(III) reduction of the high-temperature and NaCl-tolerant Shewanella xiamenensis from Changqing oilfield of China
Source: Front Microbiol. 2022 Dec 5;13:1028030. doi: 10.3389/fmicb.2022.1028030 (PMC9760863; doi:10.3389/fmicb.2022.1028030)
Supplement: Supplementary file 1 [file Data_Sheet_1.docx]

**Growth and genome-based insights of Fe(III) reduction of the high-temperature and NaCl-tolerant** ***Shewanella xiamenensis* from Changqing oilfield of China**

Jiani Yang ^1^, Dan Zhao ^1^, Tao Liu ^1, 2^, Shuang Zhang ^1^, Weidong Wang ^1, 2^, Lei Yan ^1^*, Ji-Dong Gu ^3, 4^

^1^ Heilongjiang Provincial Key Laboratory of Environmental Microbiology and Recycling of Argo-Waste in Cold Region, College of Life Science and Biotechnology, Heilongjiang Bayi Agricultural University, Daqing, 163319, PR China.

^2^ Key Laboratory of Low-carbon Green Agriculture in Northeastern China, Ministry of Agriculture and Rural Affairs P. R. China, Daqing, 163319, PR China.

^3^ Environmental Science and Engineering Research Group, Guangdong Technion - Israel Institute of Technology, 241 Daxue Road, Shantou, Guangdong 515063, PR China

^4^ Guangdong Provincial Key Laboratory of Materials and Technologies for Energy Conversion, Guangdong Technion – Israel Institute of Technology, 241 Daxue Road, Shantou, Guangdong 515063, PR China

***Corresponding author**

Lei Yan

Address: Heilongjiang Provincial Key Laboratory of Environmental Microbiology and Recycling of Argo-Waste in Cold Region, College of Life Science and Biotechnology, Heilongjiang Bayi Agricultural University, Daqing, 163319, PR China.

E-mail address: hekouyanlei@gmail.com

ORCID ID: <https://orcid.org/0000-0003-2596-6840>

**Supplementary Materials**

**Table S1.** Genes associated with NaCl resistance on genome of *S. xiamenensis* CQ-Y1

| Gene | Function | Classification |
| --- | --- | --- |
| *nhaC* | Na^+^/H^+^ antiporter NhaC | Na^+^/H^+^ antiporter |
| *nhaA* | Na^+^/H^+^ antiporter NhaA | Na^+^/H^+^ antiporter |
| *nhaC* | Na^+^/H^+^ antiporter NhaC | Na^+^/H^+^ antiporter |
| *nhaB* | Na^+^/H^+^ antiporter NhaB | Na^+^/H^+^ antiporter |
| *chaA* | Sodium exchanger protein | Na^+^/H^+^ antiporter |
| *kefB* | Kef-type K^+^ transport system%2C membrane component KefB | Na^+^/H^+^ antiporter |
| *nhaP* | NhaP-type Na^+^/H^+^ or K^+^/H^+^ antiporter | Na^+^/H^+^ antiporter |
| *kefB* | Kef-type K^+^ transport system%2C membrane component KefB | Na^+^/H^+^ antiporter |
| *araJ* | Major Facilitator Superfamily | Na^+^/H^+^ antiporter |
| *fucP* | Major Facilitator Superfamily | Na^+^/H^+^ antiporter |
| *plsC* | Major Facilitator Superfamily | Na^+^/H^+^ antiporter |
| *sul1* | Molybdate transporter of MFS superfamily | Na^+^/H^+^ antiporter |
| *araJ* | Major Facilitator Superfamily | Na^+^/H^+^ antiporter |
| *sulP* | Sulfate permease or related transporter%2C MFS superfamily | Na^+^/H^+^ antiporter |
| *gluP* | Major Facilitator Superfamily | Na^+^/H^+^ antiporter |
| *araJ* | MFS_1 like family | Na^+^/H^+^ antiporter |
| *sul1* | Sulfate permease or related transporter%2C MFS superfamily | Na^+^/H^+^ antiporter |
| *fucP* | Major Facilitator Superfamily | Na^+^/H^+^ antiporter |
| *btlA* | Major Facilitator Superfamily | Na^+^/H^+^ antiporter |
| *araJ* | Major Facilitator Superfamily | Na^+^/H^+^ antiporter |
| *gph* | MFS/sugar transport protein | Na^+^/H^+^ antiporter |
| *sul1* | Sulfate permease or related transporter%2C MFS superfamily | Na^+^/H^+^ antiporter |
| *araJ* | Major Facilitator Superfamily | Na^+^/H^+^ antiporter |
| *ptr2* | Major Facilitator Superfamily | Na^+^/H^+^ antiporter |
| *proP* | Major Facilitator Superfamily | Na^+^/H^+^ antiporter |
| *trkG*, *trkH* | Trk-type K^+^ transport system%2C membrane component | K^+^ transport system |
| *trkA* | Ion transport protein | K^+^ transport system |
| *nhaP2* | NhaP-type Na^+^/H^+^ and K^+^/H^+^ antiporter with C-terminal TrkAC and CorC domains | K^+^ transport system |
| *kdpD* | K^+^-sensing histidine kinase KdpD | K^+^ transport system |
| *proC* | Proline biosynthetic process | Intracellular compatibility of small molecules |
| *proA* | Proline biosynthetic process | Intracellular compatibility of small molecules |
| *proB* | Proline biosynthetic process | Intracellular compatibility of small molecules |
| *gltD* | NADPH-dependent glutamate synthase beta chain or related oxidoreductase | Intracellular compatibility of small molecules |
| *gltB1* | Glutamate synthase central domain | Intracellular compatibility of small molecules |
| *gltB2* | Glutamate synthase domain 2 | Intracellular compatibility of small molecules |
| *betT* | Choline-glycine betaine transporter | Intracellular compatibility of small molecules |
| *betA* | Choline dehydrogenase or related flavoprotein | Intracellular compatibility of small molecules |
| *glnA* | Glutamine synthetase | Intracellular compatibility of small molecules |

**Table S2.** Genes associated with high-temperature resistance on genome of *S. xiamenensis* CQ-Y1

| Gene | Function | Classification |
| --- | --- | --- |
| *dnaK* | Hsp70 protein | Heat shock protein |
| *hslJ* | Heat shock protein HslJ | Heat shock protein |
| *ibpA* | Hsp20 family protein | Heat shock protein |
| *groEL* | HSP60 family | Heat shock protein |
| *grpE* | Molecular chaperone GrpE (heat shock protein HSP-70) | Heat shock protein |
| *dnaK* | Molecular chaperone DnaK (HSP70) | Heat shock protein |
| *dnaJ* | DnaJ-class molecular chaperone with C-terminal Zn finger domain | Heat shock protein |
| *htpG* | Molecular chaperone%2C HSP90 family | Heat shock protein |
| *hslO* | Hsp33 protein | Heat shock protein |
| *htpX* | Heat shock protein HtpX | Heat shock protein |
| *rseA* | Anti sigma-E protein RseA%2C C-terminal domain | Heat shock protein |
| *leuA,* | Leucine biosynthetic | Thermal stability |
| *leuB* | Leucine biosynthetic | Thermal stability |
| *leuC* | Leucine biosynthetic | Thermal stability |
| *leuD* | Leucine biosynthetic | Thermal stability |
| *glnA* | Glutamine biosynthetic | Thermal stability |
| *glnA* | Glutamine synthetase | Thermal stability |
| *aceF* | Pyruvate dehydrogenase, dihydrolipoyltransacetylase component E2 | Energy metabolism |
| *aceE* | Pyruvate dehydrogenase, decarboxylase component E1 | Energy metabolism |
| *lpdA* | Lipoamide dehydrogenase, E3 component is part of three enzyme  complexes | Energy metabolism |
| *ackA* | Acetokinase family | Energy metabolism |
| *rpe* | Pentose-5-phosphate-3-epimerase | Energy metabolism |
| *cydB* | Cytochrome d ubiquinol oxidase subunit II | Energy metabolism |
| *metA* | MetA, homoserine O-succinyltransferase | Energy metabolism |
| *gapA* | GapA, glyceraldehyde-3-phosphate dehydrogenase | Energy metabolism |
| *hflB* | HflB, ATP-dependent protease | Inner membrance |
| *gmhB,* | D,D-heptose 1,7-bisphosphate phosphatase | Lipopolysaccharide biosynthesis |
| *rfaB* | Glycosyl transferases group 1 | Lipopolysaccharide biosynthesis |
| *rfaE* | ADP-heptose synthase%2C bifunctional sugar kinase/adenylyltransferase | Lipopolysaccharide biosynthesis |
| *rfaF* | Glycosyltransferase family 9 (heptosyltransferase) | Lipopolysaccharide biosynthesis |
| *yciS* | Lipopolysaccharide assembly protein A domain | Lipopolysaccharide biosynthesis |
| *yfgL* | Outer membrane assembly lipoprotein YfgL | Peptidoglycan-associated lipoprotein |
| *nlpI* | Lipoprotein NlpI | Peptidoglycan-associated lipoprotein |
| *ompA* | Outer membrane protein OmpA and related peptidoglycan-associated (lipo) proteins | Outer membrane integrity |
| *tolA* | Membrane protein TolA | Outer membrane integrity |
| *tolQ* | TolQ proton channel family | Outer membrane integrity |
| *tolR* | Biopolymer transport protein TolR | Outer membrane integrity |
| *ppiD* | PpiD, peptidyl-prolyl cis-trans isomerase | Outer membrane integrity |
| *htrB* | Integral component of membrane | Outer membrane integrity |
| *dnaQ* | DNA polymerase III%2C epsilon subunit or related 3'-5' exonuclease | DNA repair |
| *holC* | DNA polymerase III chi subunit%2C HolC | DNA repair |
| *priA* | Primosomal protein N' | DNA repair |
| *ruvC* | Crossover junction endodeoxyribonuclease RuvC | DNA repair |
| *ruvA* | Helix-hairpin-helix domain | DNA repair |
| *ruvB* | Holliday junction DNA helicase ruvB N-terminus | DNA repair |
| *iscS* | Sulfer relay system, cysteine desulfurase | tRNA modification |
| *tusE* | Sulfur relay protein | tRNA modification |
| *tusB* | Sulfur relay protein | tRNA modification |
| *tusC* | Sulfur relay protein | tRNA modification |
| *tusD* | Sulfur relay protein | tRNA modification |
| *tusA* | Sulfur carrier protein TusA | tRNA modification |
| *miaA* | tRNA dimethylallyltransferase | tRNA modification |
| *miaB* | tRNA A37 methylthiotransferase MiaB | tRNA modification |
| *miaE* | tRNA-(MS[2]IO[6]A)-hydroxylase (MiaE) | tRNA modification |
| *miaB* | tRNA A37 methylthiotransferase MiaB | tRNA modification |
| *truD* | tRNA pseudouridine synthase D (TruD) | tRNA modification |
| *truA* | tRNA pseudouridine synthase | tRNA modification |
| *truB* | tRNA pseudouridylate synthase B C-terminal domain | tRNA modification |
| *ftsJ* | FtsJ, ribosomal RNA large subunit methyltransferase | Cell division and  growth |

| **Table S3.** Genes associated with biofilm formation on genome of *S. xiamenensis* CQ-Y1 | | |
| --- | --- | --- |
| Name | Function | Classification |
| *fapA* | Flagellar assembly protein A | Flagellar |
| *fliO*, *fliOZ,* | Flagellar biosynthesis protein%2C FliO | Flagellar |
| *fliA* | Sigma-70%2C region 4 | Flagellar |
| *fliD* | Flagellin hook IN motif | Flagellar |
| *fliF* | Flagellar M-ring protein C-terminal | Flagellar |
| *,fliG* | Flagellar motor switch protein FliG | Flagellar |
| *fliI* | Flagellar biosynthesis/type III secretory pathway ATPase FliI | Flagellar |
| *flgL*, *fliC* | Bacterial flagellin C-terminal helical region | Flagellar |
| *fliP* | Flagellar biosynthesis protein FliP | Flagellar |
| *fliN* | Type III flagellar switch regulator (C-ring) FliN C-term | Flagellar |
| *fliR* | Flagellar biosynthetic protein FliR | Flagellar |
| *fliQ* | Flagellar biosynthetic protein FliQ | Flagellar |
| *fliE* | Flagellar hook-basal body complex protein FliE | Flagellar |
| *fliJ* | Flagellar FliJ protein | Flagellar |
| *fliH* | Flagellar assembly protein FliH | Flagellar |
| *fliK* | Flagellar hook-length control protein FliK | Flagellar |
| *fliM* | Flagellar motor switch protein FliM | Flagellar |
| *fliS* | Flagellar protein FliS | Flagellar |
| *,fliL* | Flagellar basal body-associated protein FliL | Flagellar |
| *flhF* | flagellar biosynthesis protein FlhF | Flagellar |
| *flhA* | flagellar biosynthesis protein FlhA | Flagellar |
| *flhB, flhB, flhB* | flagellar biosynthetic protein FlhB | Flagellar |
| *pilT* | Twitching motility protein | Flagellar |
| *hfq* | Hfq protein | Quorum sensing |
| *luxS* | Quorum sensing;S-ribosylhomocysteine lyase activity;iron ion binding | Quorum sensing |
| *cpsB*, *manC, cpsB* | biosynthetic process | EPS |
| *gumC* | G-rich domain on putative tyrosine kinase | EPS |
| *algH* | Putative transcriptional regulator%2C AlgH/UPF0301 family | EPS |
| *galU*, *galF* | UTP-glucose-1-phosphate uridylyltransferase | EPS |
| *galM* | Galactose mutarotase or related enzyme | EPS |
| *galK* | Galactokinase galactose-binding signature | EPS |
| *galE* | Galactose metabolic process;UDP-glucose 4-epimerase activity | EPS |

**Table S4.** Genes associated with Fe(III) reduction on genome of *S. xiamenensis* CQ-Y1

| Gene | Function | Classification |
| --- | --- | --- |
| *fur* | Ferric uptake regulator family | c-Type Cytochromes |
| *coxB*, *cyoA*, *coxB, ctaC* | Cytochrome c oxidase subunit II | c-Type Cytochromes |
| *ctaD*, *cyoB*, *coxA, ctaD* | Cytochrome c oxidase subunit I | c-Type Cytochromes |
| *ctaG* | Cytochrome c oxidase assembly protein CtaG/Cox11 | c-Type Cytochromes |
| *cyoC*, *coxC*, *ctaE* | Cytochrome c oxidase assembly protein CtaG/Cox11 | c-Type Cytochromes |
| *ctaA*, *cox15*, *ctaA* | Cytochrome C and Quinol oxidase polypeptide I | c-Type Cytochromes |
| *cyoB* | Cytochrome C and Quinol oxidase polypeptide I | c-Type Cytochromes |
| *cyoC* | Cytochrome c oxidase subunit III | c-Type Cytochromes |
| *cyoD* | Prokaryotic Cytochrome C oxidase subunit IV | c-Type Cytochromes |
| *cytC553* | Cytochrome c | c-Type Cytochromes |
| *ccoG* | Cytochrome c oxidase accessory protein CcoG | c-Type Cytochromes |
| *ccmE* | Cytochrome c maturation protein CcmE | c-Type Cytochromes |
| *ccmC* | Cytochrome C assembly protein | c-Type Cytochromes |
| *ccmB* | CcmB protein | c-Type Cytochromes |
| *ccmA* | Cytochrome c biogenesis heme-transporting ATPase CcmA | c-Type Cytochromes |
| *cytC5* | Cytochrome c5 family protein | c-Type Cytochromes |
| *ccmI*, *nrfG*, *ccmH* | Cytochrome c-type biogenesis protein CcmI | c-Type Cytochromes |
| *nrfF*, *ccmH* | Cytochrome C biogenesis protein | c-Type Cytochromes |
| *cytC553* | C-type cytochrome | c-Type Cytochromes |
| *tsdA* | Cytochrome c | c-Type Cytochromes |
| *nrfA* | Cytochrome c552 | c-Type Cytochromes |
| *sdhA*, *frdA* | Cytochrome c3 | c-Type Cytochromes |
| *norB* | Cytochrome C and Quinol oxidase polypeptide I | c-Type Cytochromes |
| *cytC556* | Cytochrome c556 | c-Type Cytochromes |
| *ccoN* | Cytochrome C and Quinol oxidase polypeptide I | c-Type Cytochromes |
| *ccoO* | Cytochrome C oxidase%2C mono-heme subunit/FixO | c-Type Cytochromes |
| *ccoP* | Cytochrome c oxidase%2C cbb3-type%2C subunit III | c-Type Cytochromes |
| *dsbD* | Cytochrome C biogenesis protein transmembrane region | c-Type Cytochromes |
| *napC* | Cytochrome c-type protein NapC | c-Type Cytochromes |
| *napB* | Cytochrome c-type protein NapB | c-Type Cytochromes |
| *sdhA*, *frdA* | Cytochrome c3 | c-Type Cytochromes |
| *omcA*, *mtrC* | OmcA/MtrC family decaheme c-type cytochrome | OmcA/MtrC |
| *mtrB* | MtrB/PioB family decaheme-associated outer membrane protein | MtrB |
| *ribE*, *ribC* | Riboflavin synthase alpha chain | Riboflavin |
| *ribH*, *ribE* | Riboflavin synthase alpha chain | Riboflavin |
| *ribA* | Riboflavin biosynthetic process;GTP cyclohydrolase II activity | Riboflavin |
| *ribB* | Riboflavin biosynthetic process;GTP cyclohydrolase II activity | Riboflavin |
| *ribB*, *ribBA* | Riboflavin biosynthetic process;GTP cyclohydrolase II activity | Riboflavin |
| *ribD1*, *ribD* | Riboflavin biosynthesis protein RibD%2C pyrimidine deaminase domain | Riboflavin |
| *ribF* | FAD synthetase | Riboflavin |
| *IucA* | Siderophore synthetase component IucA/IucC/SbnC | Siderophore |
| *fhuE*, *fpvA*, *fptA* | Transport;outer membrane;membrane;siderophore transmembrane transporter activity | Siderophore |
| *fhuE* | Transport;outer membrane;membrane;siderophore transmembrane transporter activity | Siderophore |
| *fhuF* | Siderophore-iron reductase FhuF | Siderophore |

**Table S5.** Quantification of biofilm formation at different NaCl concentrations

| NaCl Concentrations（% w/v） | Cut-Off Value Calculation | Mean of OD Values | Biofilm Formation Abilities |
| --- | --- | --- | --- |
| 0 | 2×Ac < A ≤ 4×Ac | 0.56 < OD ≤ 1.12 | Moderate |
| 2 | 2×Ac < A ≤ 4×Ac | 0.56 < OD ≤ 1.12 | Moderate |
| 4 | 2×Ac < A ≤ 4×Ac | 0.56 < OD ≤ 1.12 | Moderate |
| 6 | 2×Ac < A ≤ 4×Ac | 0.56 < OD ≤ 1.12 | Moderate |
| 8 | (4**×**Ac) < A | OD > 1.12 | Strong |
| 10 | (4**×**Ac) < A | OD > 1.12 | Strong |
| 12 | (4**×**Ac) < A | OD > 1.12 | Strong |

**Table S6.** Quantification of biofilm formation at different temperatures

| Temperatures (°C) | Cut-Off Value Calculation | Mean of OD Values | Biofilm Formation Abilities |
| --- | --- | --- | --- |
| 30 | 2×Ac < A ≤ 4×Ac | 0.56 < OD ≤ 1.12 | Moderate |
| 35 | 2×Ac < A ≤ 4×Ac | 0.56 < OD ≤ 1.12 | Moderate |
| 40 | 2×Ac < A ≤ 4×Ac | 0.56 < OD ≤ 1.12 | Moderate |
| 45 | Ac < A ≤ 2×Ac | 0.28 < OD ≤ 0.56 | Low |
| 50 | Ac < A ≤ 2×Ac | 0.28 < OD ≤ 0.56 | Low |

**Table S7.** Comparison of partial genes related to Fe(III) reduction, NaCl and high temperature resistance from the genomes of *Shewanella* spp..

| Characteristic | Gene | *S. xiamenensis* CQ-Y1 | *S. algae* ATCC 49138 | *S. oneidensis* MR-1 | *S. frigidimarina*  NCIMB 400 | *S. putrefaciens* ATCC 8071 |
| --- | --- | --- | --- | --- | --- | --- |
| NaCl resistance | *betA* | + | + | - | + | - |
|  | *betT* | + | - | - | - | - |
|  | *nhaA* | + | + | + | + | + |
|  | *nhaB* | + | + | + | + | + |
|  | *nhaC* | + | + | + | + | + |
|  | *trkA* | + | + | + | + | + |
|  | *trkH* | + | + | + | + | + |
|  | *kdpD* | + | - | + | - | - |
|  | *proA* | + | - | + | - | - |
|  | *gltD* | + | - | + | - | - |
| High-temperature resistance | *hslJ* | + | - | - | - | - |
|  | *ibpA* | + | - | + | - | - |
|  | *rfaB* | + | - | - | - | - |
|  | *htrB* | + | - | - | - | - |
|  | *ftsJ* | + | - | - | - | - |
|  | *gapA* | + | - | + | - | - |
|  | *hflB* | + | - | - | - | - |
|  | *metA* | + | + | + | - | + |
|  | *holC* | + | - | + | - | - |
|  | *ppiD* | + | - | + | - | - |
| Fe(III) reduction | *ribA* | + | + | + | + | + |
|  | *ribB* | + | + | + | + | + |
|  | *ribM* | + | - | - | - | - |
|  | *ribH* | + | - | - | - | + |
|  | *omcA* | + | + | - | + | + |
|  | *mtrC* | + | + | + | + | + |
|  | *mtrA* | + | - | + | - | - |
|  | *mtrB* | + | + | + | + | + |
|  | *cymA* | + | - | + | - | + |
|  | *fccA* | + | + | + | - | + |

+ Positive reaction

**-** Negative reaction

**Table S8.** Comparison of Fe(III) reduction ratios, NaCl and high temperature resistance of *S. xiamenensis* CQ-Y1 with those of other *Shewanella* spp..

| Species | Fe(III) reduction | | Maximum temperature tolerance (^o^C) | Maximum NaCl tolerance (% w/v) | Reference |
| --- | --- | --- | --- | --- | --- |
|  | Substrate | Ratio (%) |  |  |  |
| *S. xiamenensis* CQ-Y1 | Fe(OH)_3_ | 70.10 | 45 | 10 | This study |
| *S. algae* ATCC 49138 | - | - | 42 | 6.5 | Khashe and Janda, 1998 |
| *S. oneidensis*  MR-1 | γ-FeOOH | 75.9 | 40 | 8 | O'Loughlin et al., 2007; Wu et al., 2012 |
| *S. frigidimarina* ACAM 591^T^ | - | - | 27 | 8 | Zeng et al., 2010 |
| *S. putrefaciens* ATCC 8071 | γ-FeOOH | 74.3 | 35 | 6 | O'Loughlin et al., 2007; Khashe and Janda et al., 1998; Vogel et al., 1997 |

- Not provided


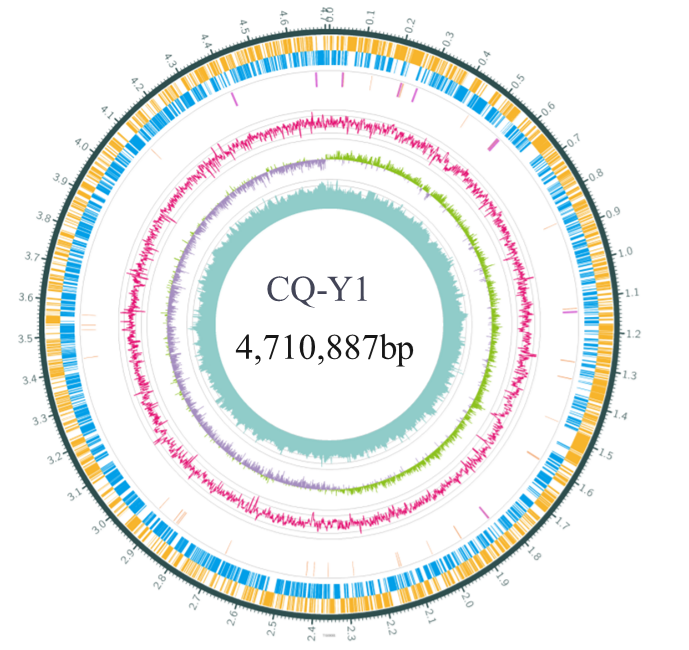
**Figure S1.** Circular genome map of the *S. xiamenensis* CQ-Y1. From outer to inner, genes on direct strand, genes on complementary strand, tRNAs (orange), rRNAs (purple), CRISPR (blue) and genomic island (green), GC-skew and sequencing depth are displayed

**Figure S2.** Crystal violet staining micrographs (A, C) and quantification (B, D) of biofilm formed at different NaCl concentrations (A, B) and temperatures (C, D).


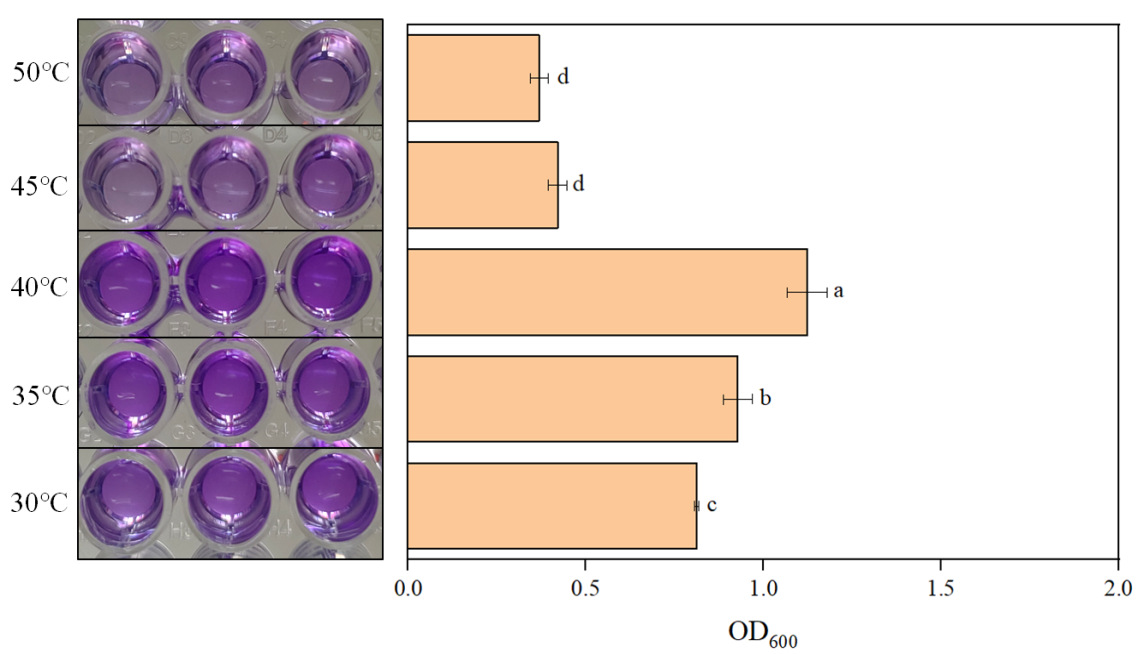


C

D


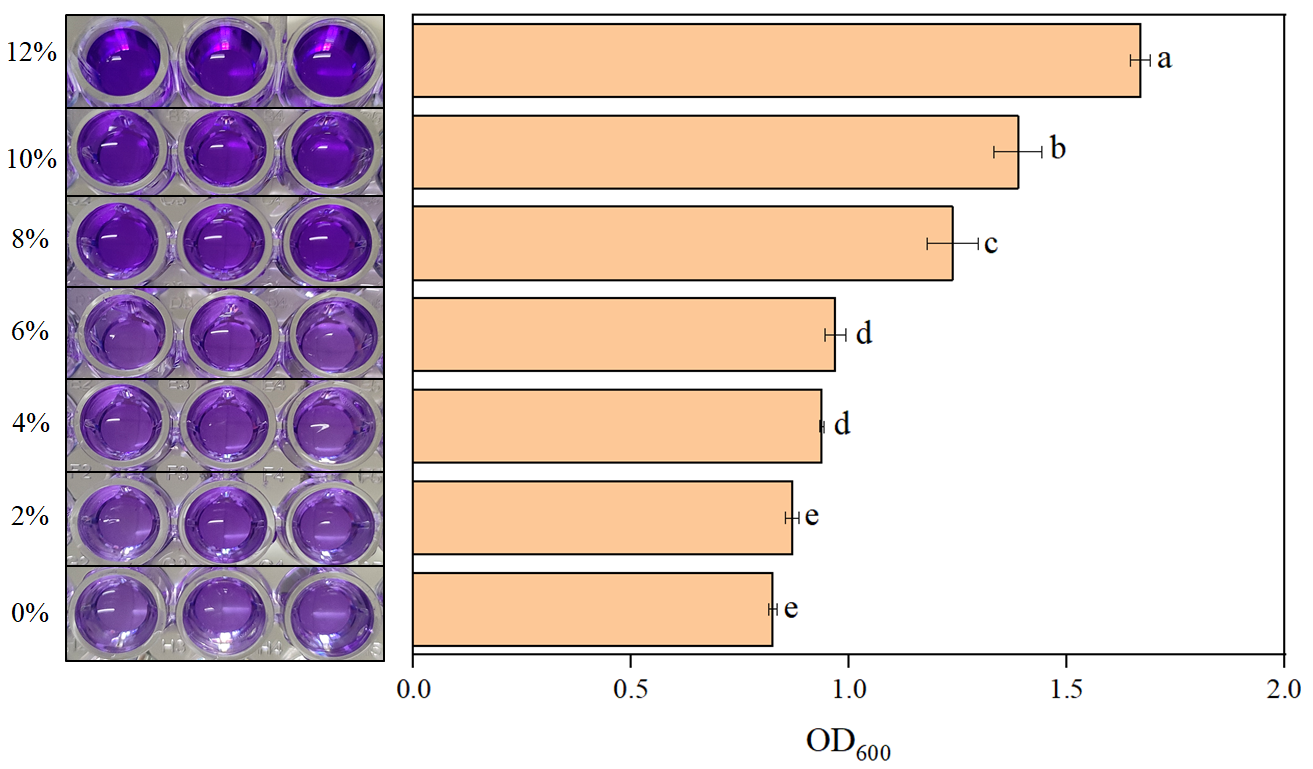


A

B

**Figure S3.** The relationship of bacterial growth with NaCl at different temperatures (A, 30 °C; B, 40 °C; C, 45 °C), and culture temperature (D).


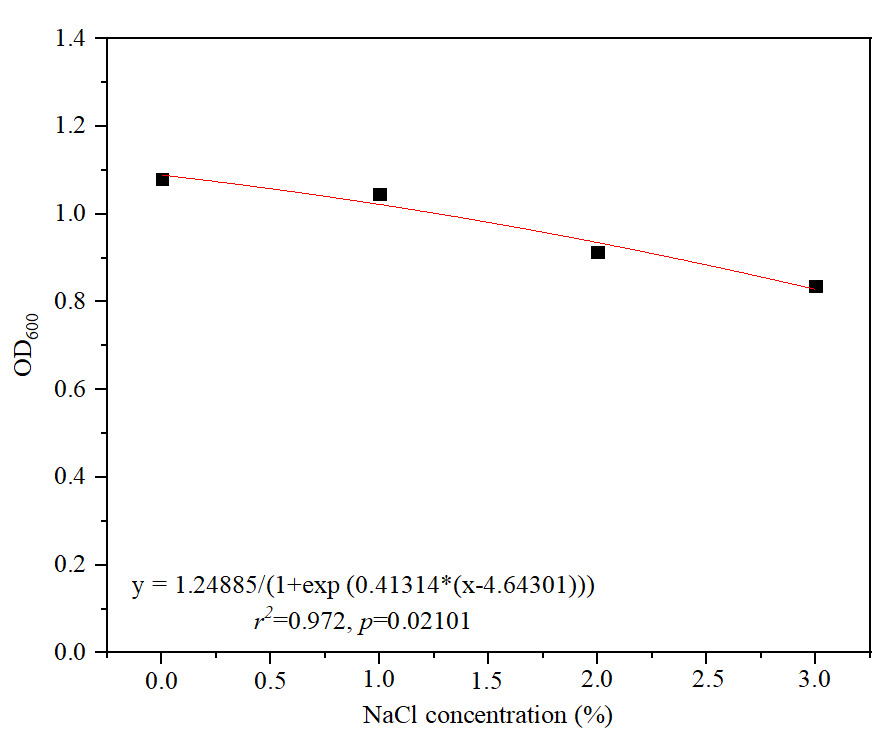

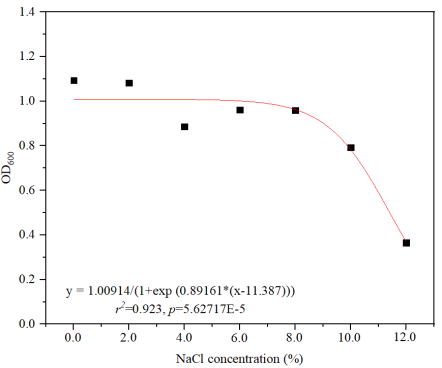


A

B

C

D


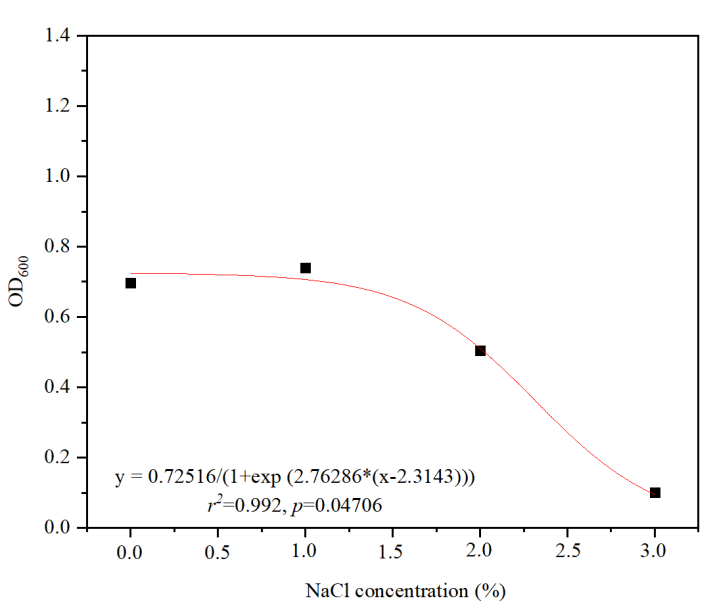

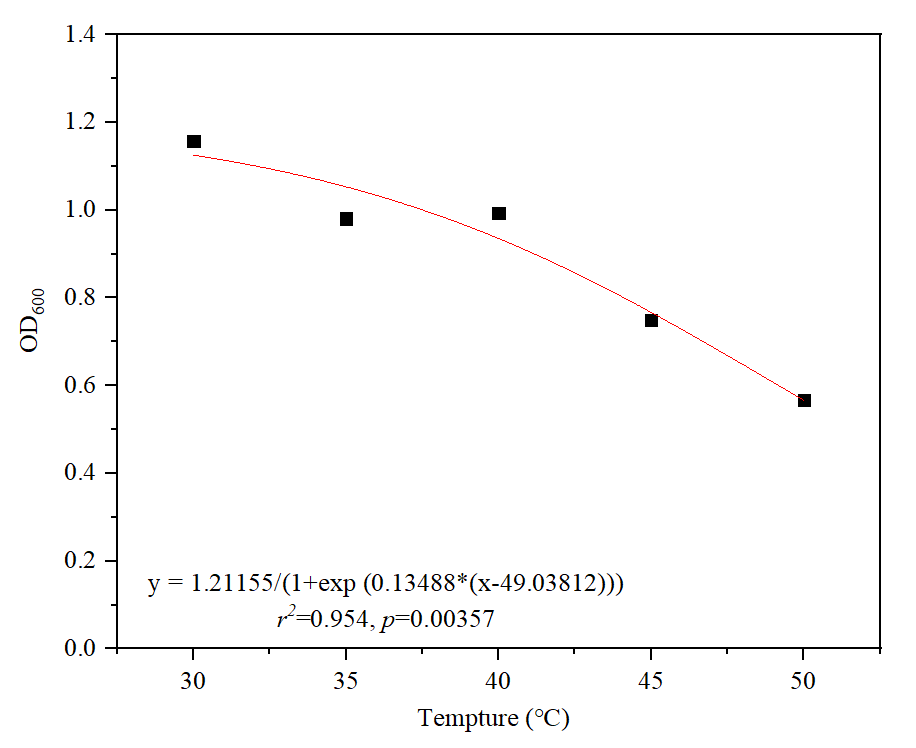


**Figure S4.** The relationship of Fe(III) reduction with NaCl at different temperatures (A, 30 °C; B, 40 °C; C, 45 °C), and culture temperature (D).


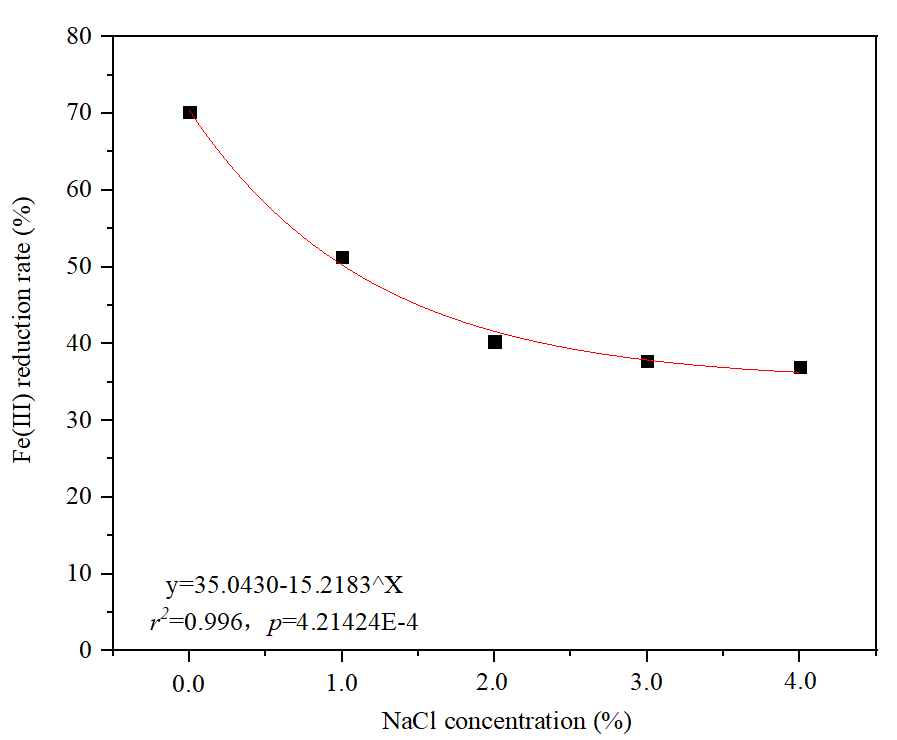

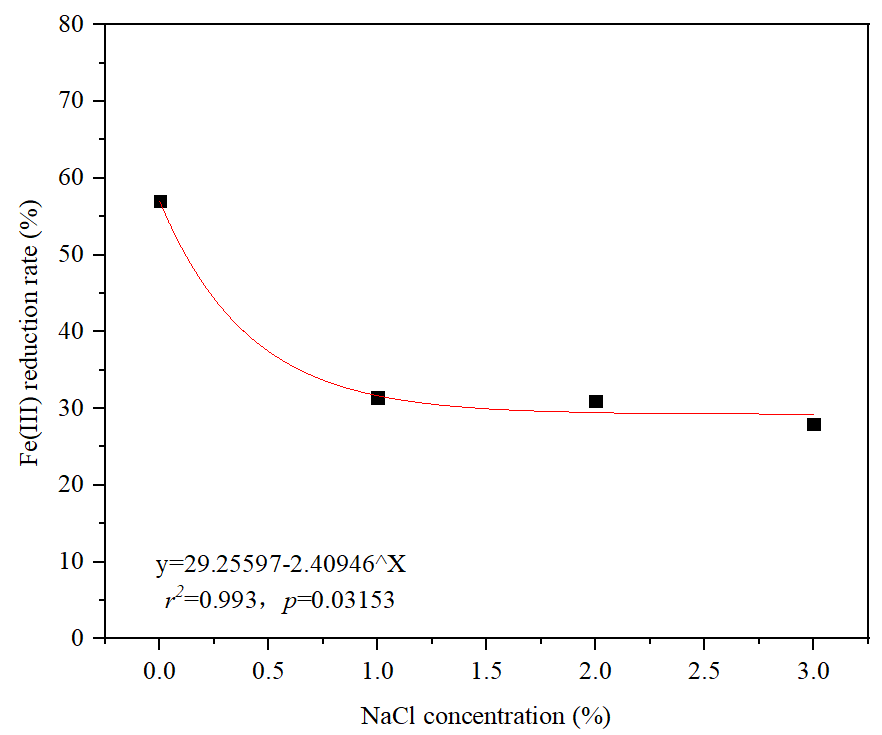

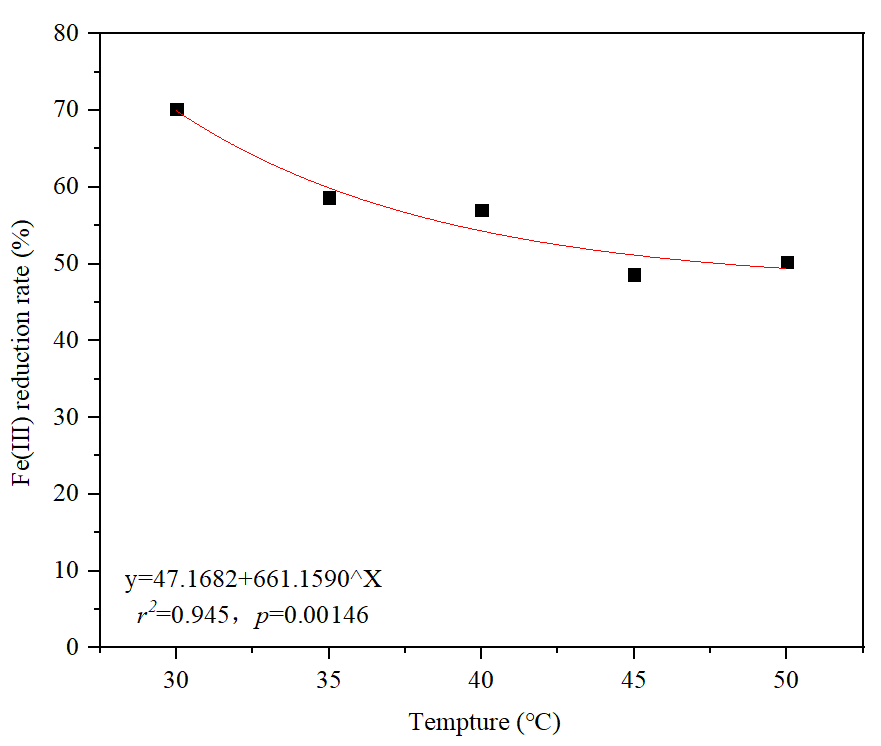

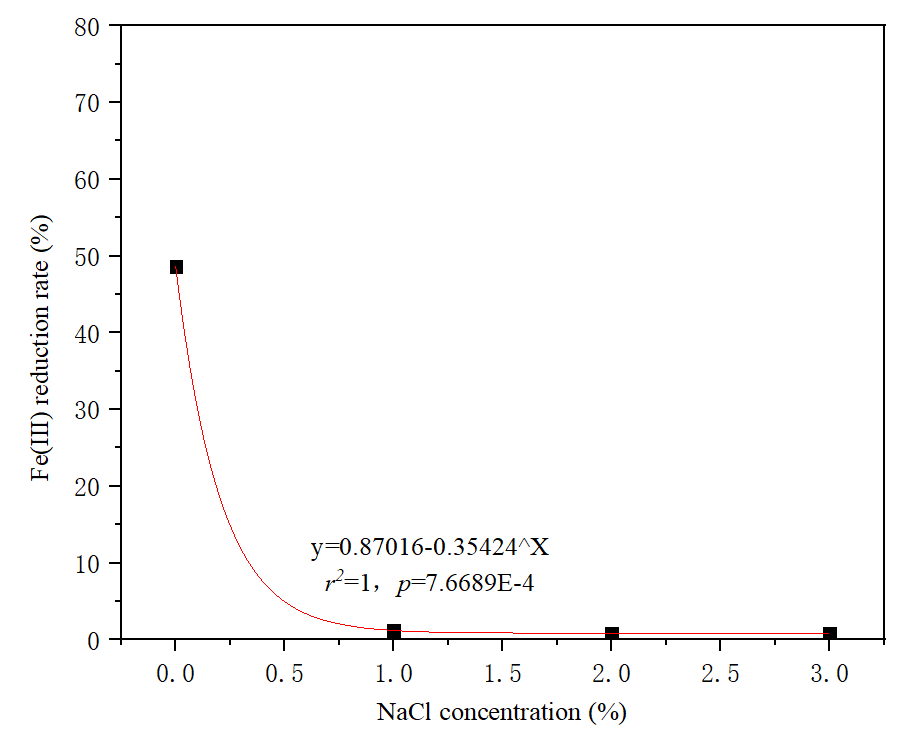


A

B

C

D
